# Supplementary figures and images for: A Flexible, Efficient Binomial Mixed Model for Identifying Differential DNA Methylation in Bisulfite Sequencing Data
Source: PLoS Genet. 2015 Nov 24;11(11):e1005650. doi: 10.1371/journal.pgen.1005650 (PMC4657956; doi:10.1371/journal.pgen.1005650)

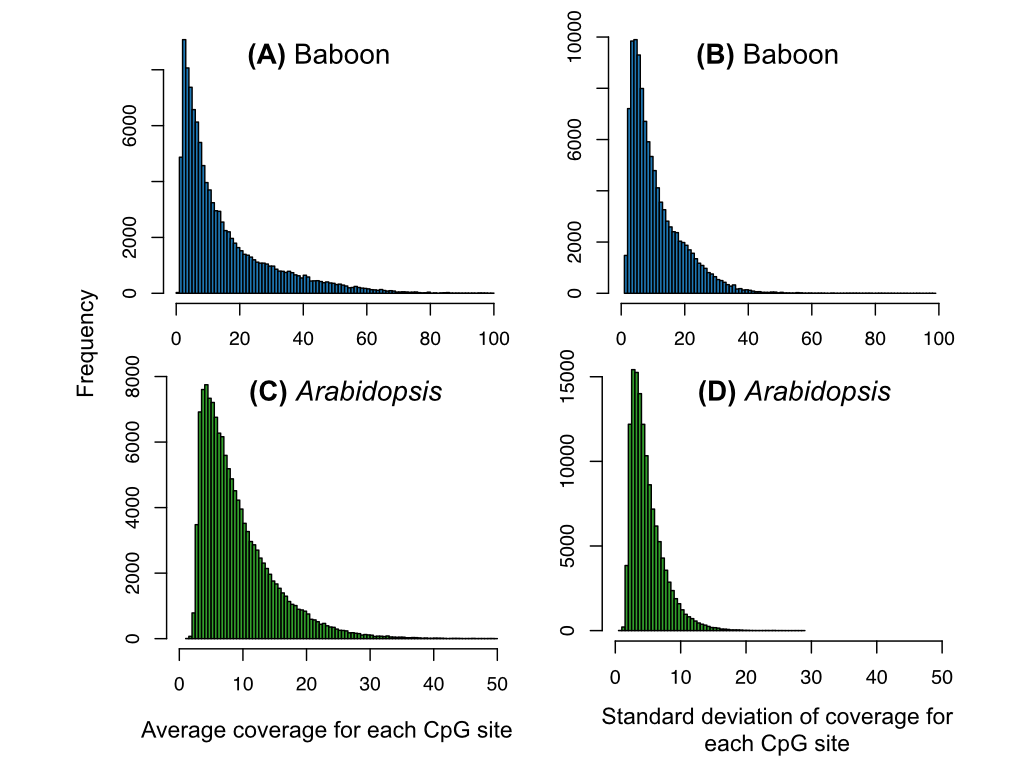

Supplement: S1 Fig — For each CpG site represented in each data set (n = 433,871 for baboon and n = 830,676 for Arabidopsis), we calculated the mean site-specific coverage across individuals, as well as the standard deviation of coverage values for those sites. The distribution of these values are are shown for the baboon RRBS dataset (A-B, in blue) and the Arabidopis WGBS dataset (C-D, in green). Average coverage values are depicted in A and C, and coverage standard deviation values are depicted in B and D. (TIFF) [file pgen.1005650.s001.tiff]

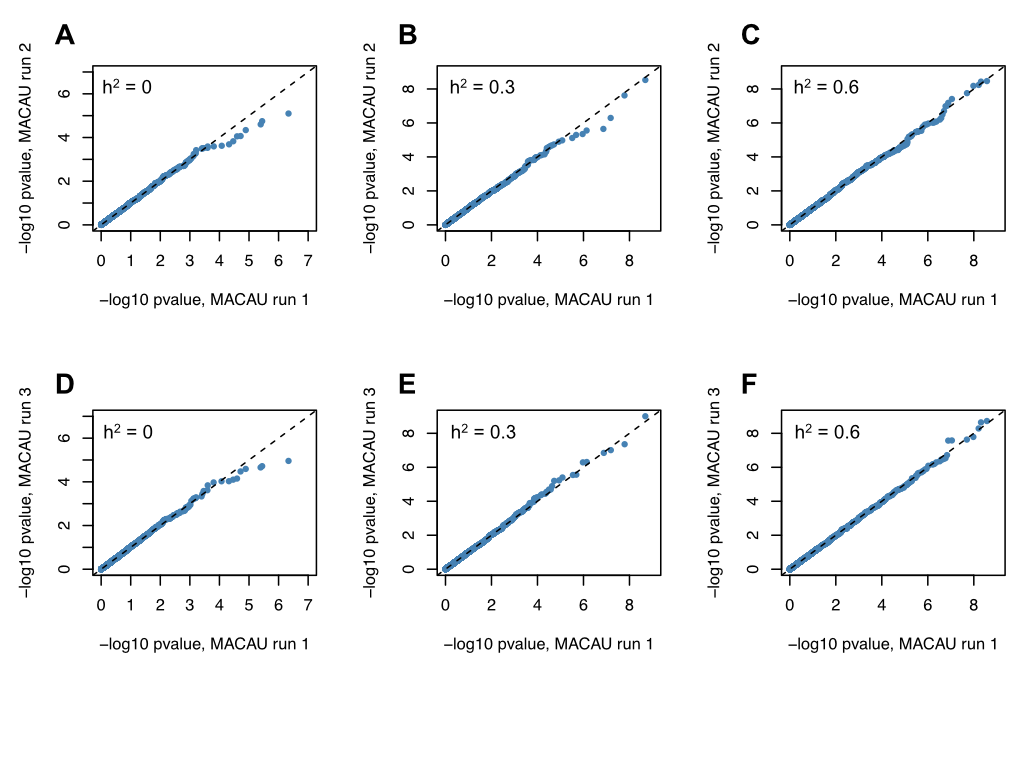

Supplement: S2 Fig — QQ-plots comparing the p-value distributions for 3 independent runs of MACAU on the same data sets, with different simulated heritability values (Panels A, D—h2 = 0; Panels B, E—h2 = 0.3; Panels C, F—h2 = 0.6). Pairwise correlations between each independent run were R > 0.95 for h2 = 0:,R > 0.97 for h2 = 0.3; and R > 0.98 for h2 = 0.6. Distributions shown are for analyses of simulated secondary dormancy effects on DNA methylation levels in the Arabidopsis data set (4000 sites, n = 24 accessions). (TIFF) [file pgen.1005650.s002.tiff]

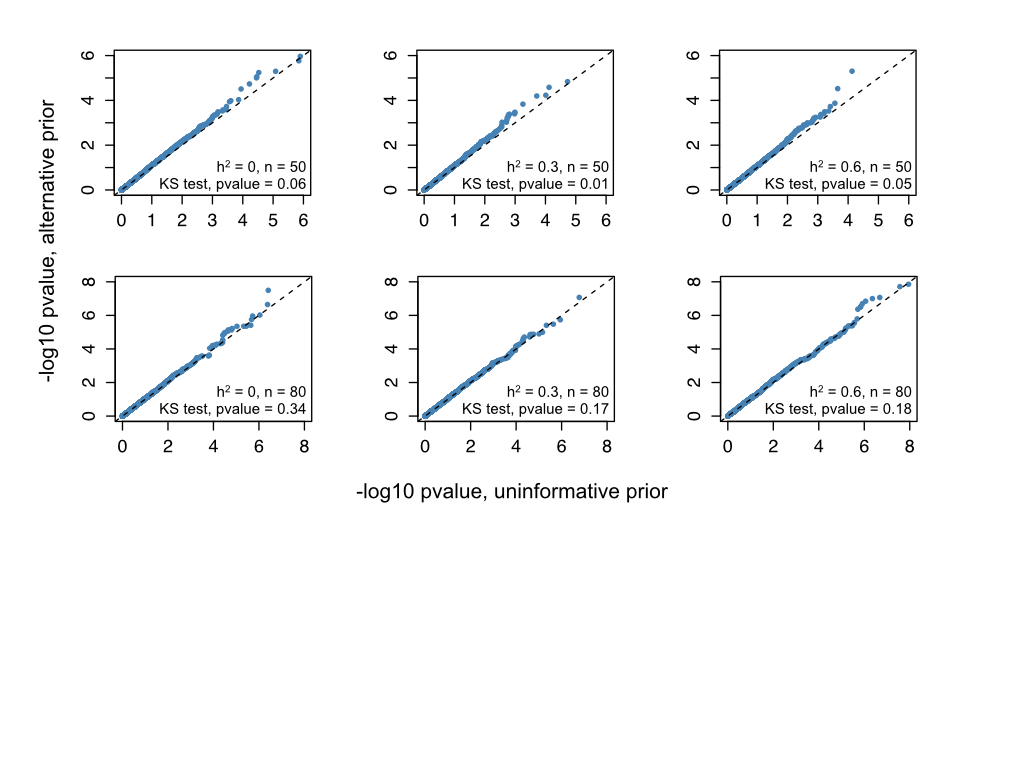

Supplement: S3 Fig — QQ-plots comparing the results from MACAU implemented with an uninformative prior (σ2 ~ U(0,1), as in the main text, x-axis) versus an alternative prior (log(σ2) ~ U(0,1), y-axis). All analyses tested for age effects on DNA methylation levels in a simulated baboon data (based on properties of the real baboon RRBS data and age information). Sample sizes and heritabilities are shown on each plot, as are the results from a Kolmogorov-Smirnov test comparing the two distributions represented in each plot. In all cases, the simulated percent variance explained by age was set to 10%. The number of age-associated sites detected in each analysis were identical for all simulations where n = 80 (10% empirical FDR), and very similar when n = 50 (0.4–0.8% more age-associated sites were detected with the alternative prior than with the uninformative prior). (TIFF) [file pgen.1005650.s003.tiff]

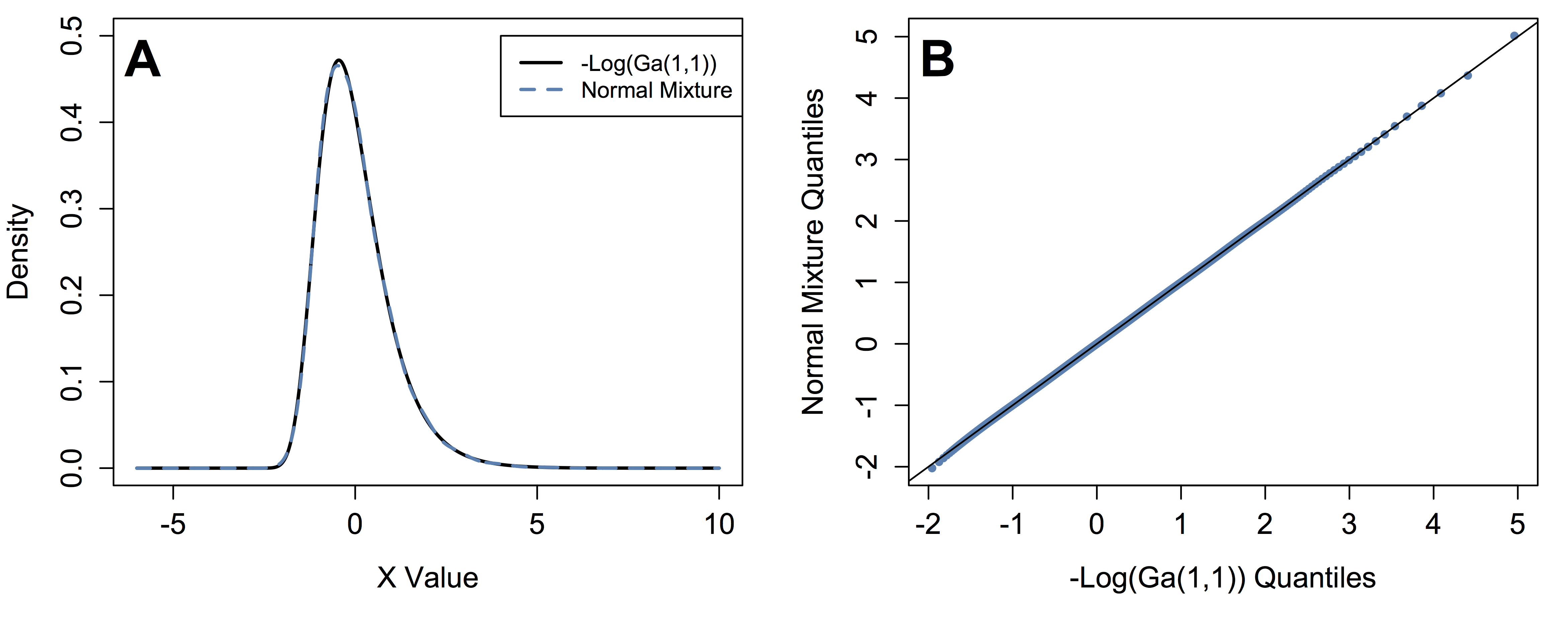

Supplement: S4 Fig — (A) Density plot and (B) quantile-quantile plots demonstrating that the normal mixture approximation approximates–log(Ga(r, 1)) well even in the most difficult case when r = 1. (TIFF) [file pgen.1005650.s004.tiff]

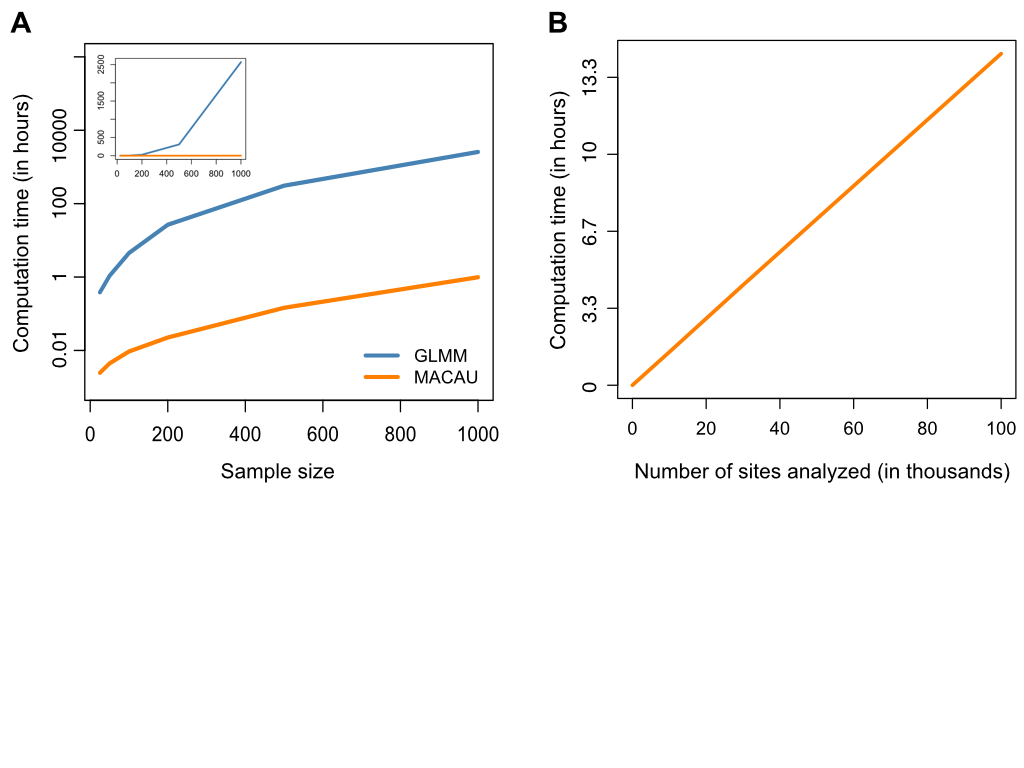

Supplement: S5 Fig — (A) Computation time (in hours) is plotted for datasets containing varying numbers of individuals, but each containing 100 sites. Computation time is plotted on a log10 scale in the main plot, and on a traditional scale in the inset. (B) Computation time (in hours) is plotted for a dataset containing 150 individuals, but varying numbers of sites (in thousands) as noted on the x-axis. All computation was performed on a single core of an Intel Xeon L5420 2.50 GHz processor. (TIFF) [file pgen.1005650.s005.tiff]

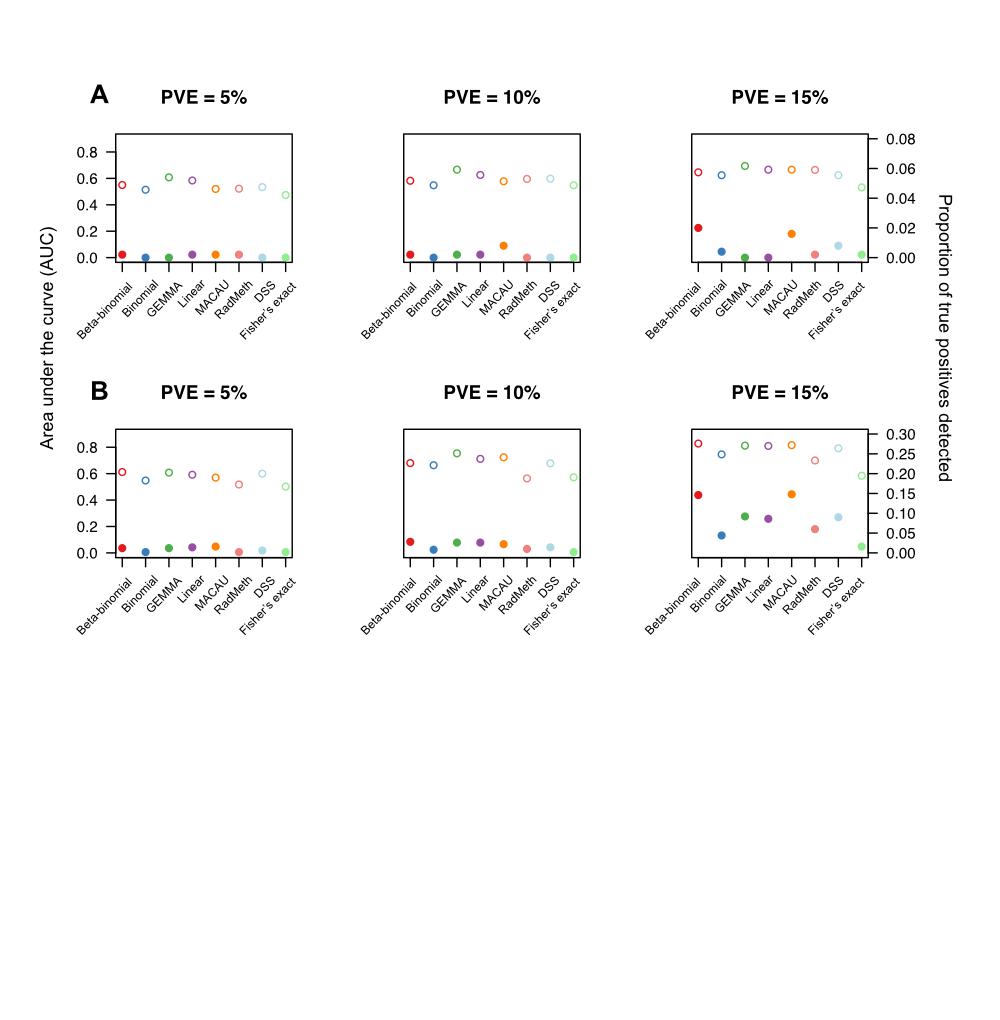

Supplement: S6 Fig — To include methods that can only analyze categorical differences in DNA methylation levels between two groups, we binarized age values in our simulated RRBS datasets (individuals below median age = young versus individuals above median age = old). We compared the AUC of each method (open circles), as well as their ability to detect true positives at a 10% FDR (closed circles). For these comparisons, we used simulated datasets with a fixed h2 of 0 (n = 5000 sites including 500 true positives and 4500 true negatives; percent variance explained by age varies as noted in the panel headings). Results for simulations with (A) n = 50 or (B) n = 80 individuals are plotted below. Note that the right-hand y axis for the proportion of true positives detected varies depending on sample size. (TIFF) [file pgen.1005650.s006.tiff]

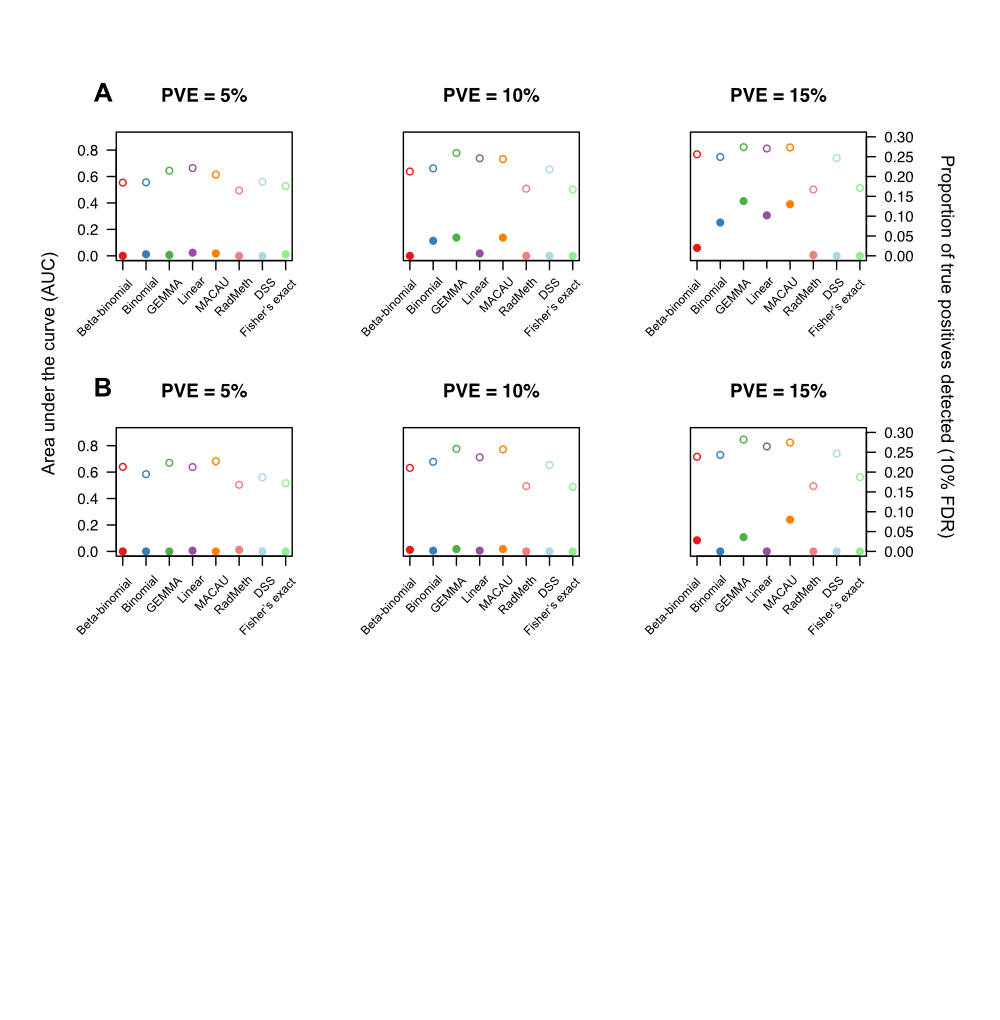

Supplement: S7 Fig — To include methods that can only analyze categorical differences in DNA methylation levels between two groups, we binarized age values in our simulated RRBS datasets (individuals below median age = young versus individuals above median age = old). We compared the AUC of each method (open circles), as well as their ability to detect true positives at a 10% FDR (closed circles). For these comparisons, we used simulated datasets with a fixed sample size of 80 (n = 5000 sites including 500 true positives and 4500 true negatives; percent variance explained by age varies as noted in the panel headings). Results for simulations with (A) h2 = 0.3 or (B) h2 = 0.6 are plotted below. (TIFF) [file pgen.1005650.s007.tiff]

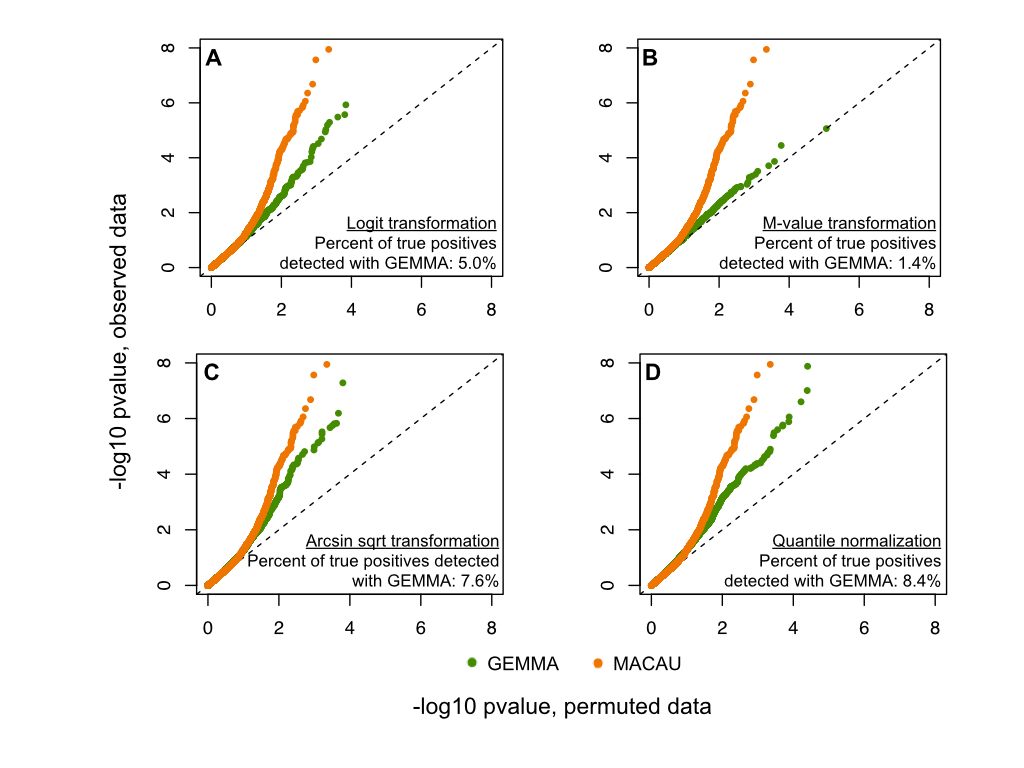

Supplement: S8 Fig — We performed four different transformations on simulated baboon bisulfite sequencing count data (n = 5000 sites including 500 true positives and 4500 true negatives; percent variance explained by age = 10%; sample size = 80, h2 = 0.6). Below, we use QQ-plots to compare the distribution of p-values produced by GEMMA (operating on the transformed data) versus MACAU (analyzing the raw count data). In all panels, the observed p-values are plotted against quantiles for the distribution of p-values obtained from running each method (MACAU or GEMMA, respectively) on permuted data. We also note the proportion of simulated true positives detected by each approach (for comparison, MACAU detects 20.6% of simulated true positives in the same dataset). (TIFF) [file pgen.1005650.s008.tiff]

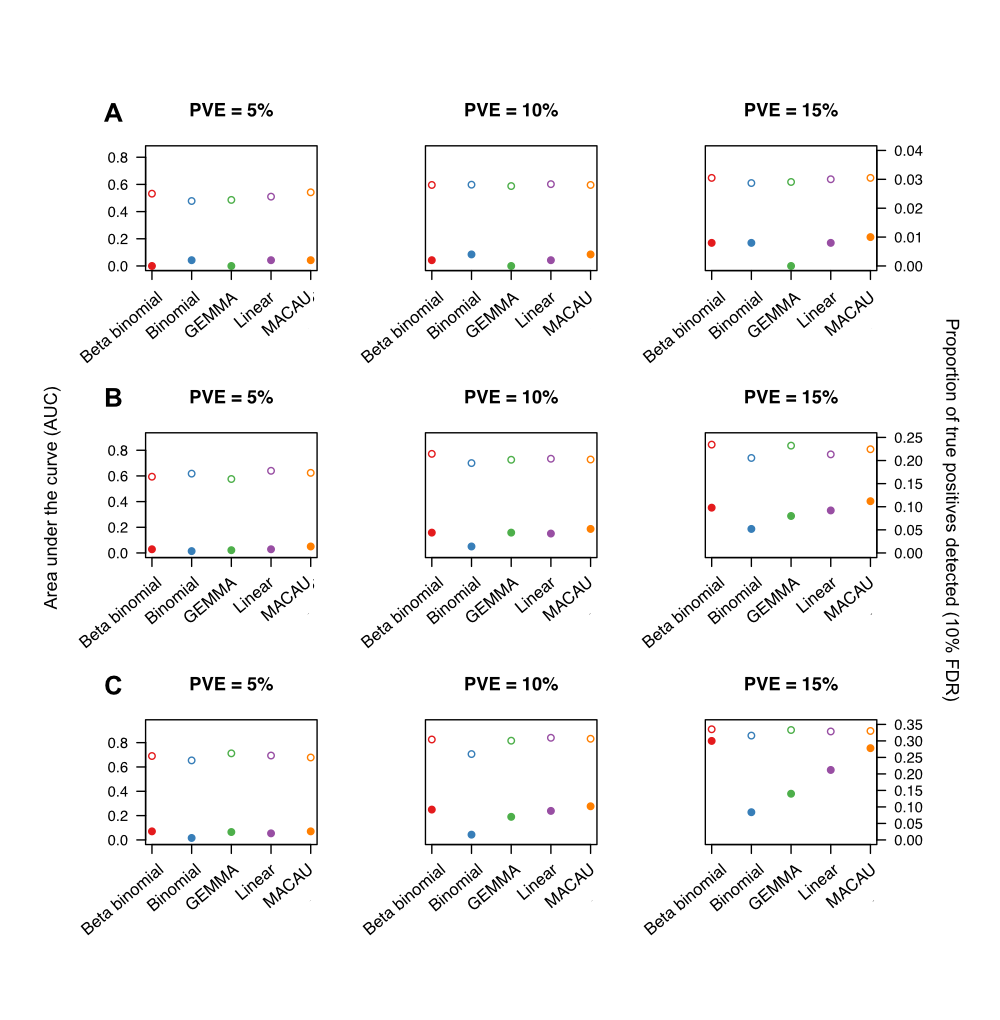

Supplement: S9 Fig — We compared the AUC of each method (open circles) and their ability to detect true positives at a 10% FDR (closed circles). We did so using simulated data sets (n = 5000 sites including 500 true positives and 4500 true negatives; percent variance explained by age varies as noted in the panel headings). For all simulations shown below, h2 was set to 0. (A) Results for simulations with n = 20 individuals; (B) with n = 50 individuals; and (C) with n = 80 individuals. Note that the right-hand y axis for the proportion of true positives detected varies depending on sample size. (TIFF) [file pgen.1005650.s009.tiff]

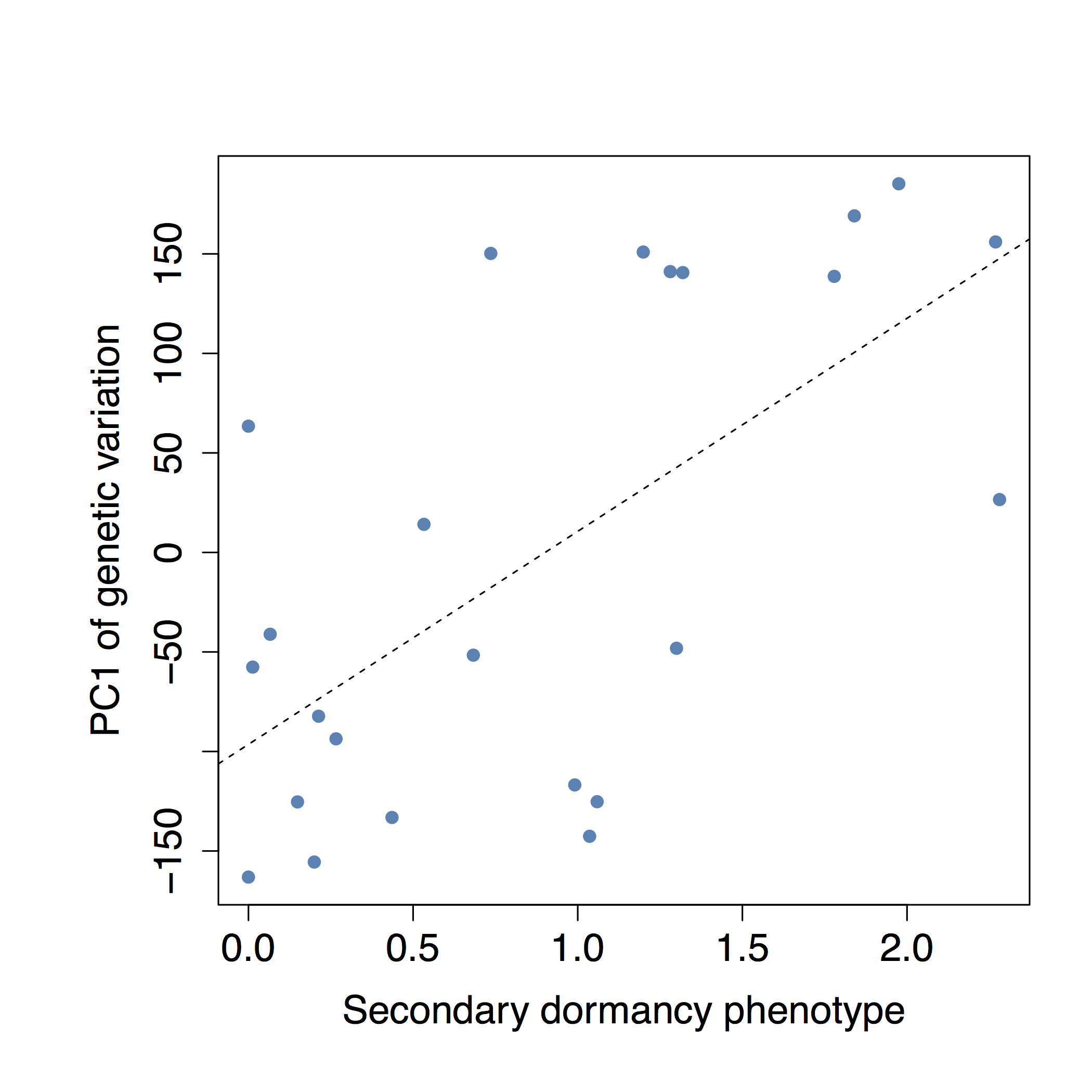

Supplement: S10 Fig — Principal components analysis on 188,093 genotyped sites with minor allele frequency >5% reveals that genetic background is correlated with secondary dormancy values. The correlation between the secondary dormancy phenotype values and the first principal component of the genetic relatedness matrix is R2 = 0.38, p = 7.84 x 10^−4^ (n = 24). The first principal component (PC1) explains 8.5% of the genetic variance in the data set. (TIFF) [file pgen.1005650.s010.tiff]

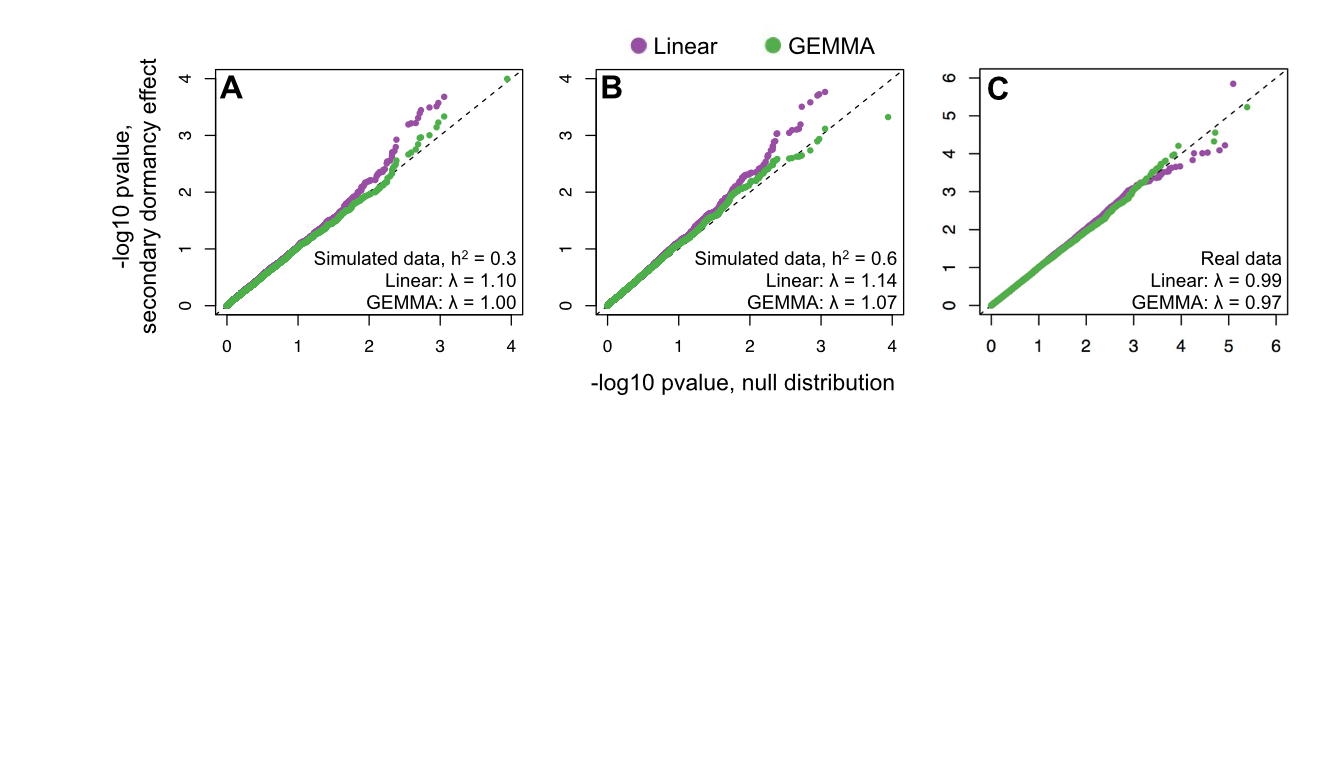

Supplement: S11 Fig — (A, B) The distribution of p-values for 4000 simulated true negative sites (n = 24 accessions; effect of secondary dormancy on DNA methylation levels = 0). For each simulation, h2 was set to 0.3 (A) or 0.6 (B). Simulated data were analyzed with a linear model or GEMMA, and compared against the expected uniform distribution. (C) QQ-plots comparing the p-value distributions for (i) a model testing for effects of secondary dormancy on DNA methylation levels in real WGBS data, plotted on the y-axis; and (ii) the same model when the secondary dormancy values were permuted across individuals, plotted on the x-axis. Here, the lack of inflated test statistics in the case of the linear model is likely due to the model’s low power (see S12B Fig, for n = 25). The genomic control factor, λ, is shown for each set of results. (TIFF) [file pgen.1005650.s011.tiff]

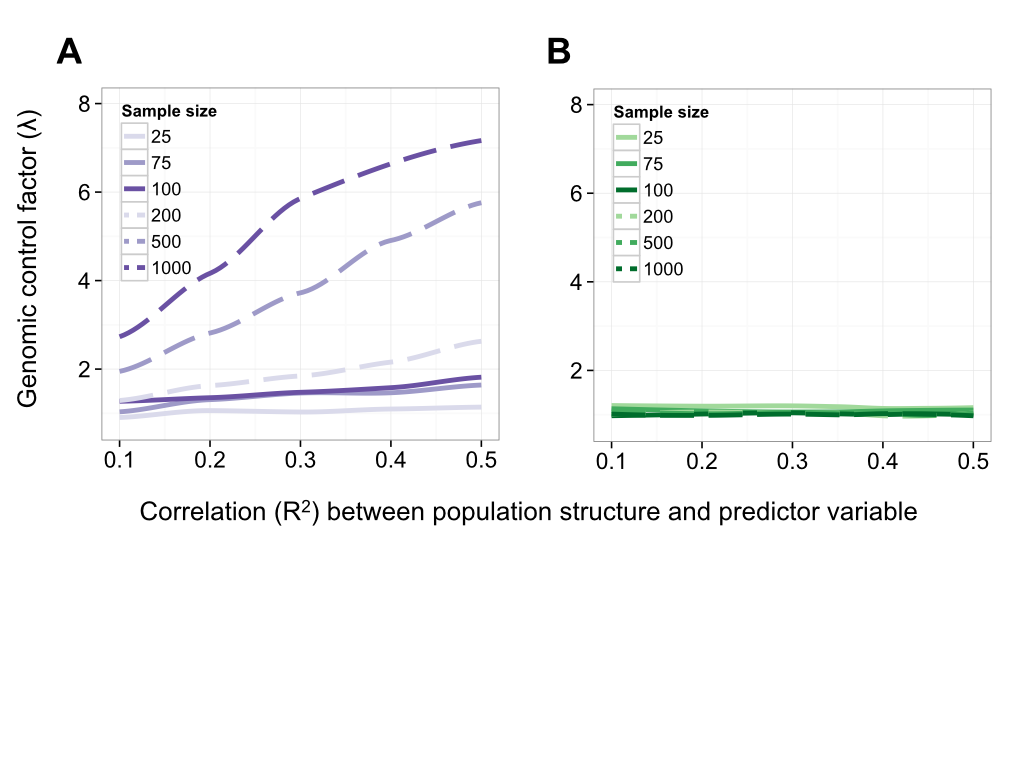

Supplement: S12 Fig — Genomic control factor when simulated datasets (n = 5000 sites per dataset; h2 = 0.6) were analyzed with either (A) a linear model or (B) a linear mixed model implemented in GEMMA. The correlation between the simulated predictor variable and the first principal component of genome-wide genotype data is plotted on the x-axis. (TIFF) [file pgen.1005650.s012.tiff]

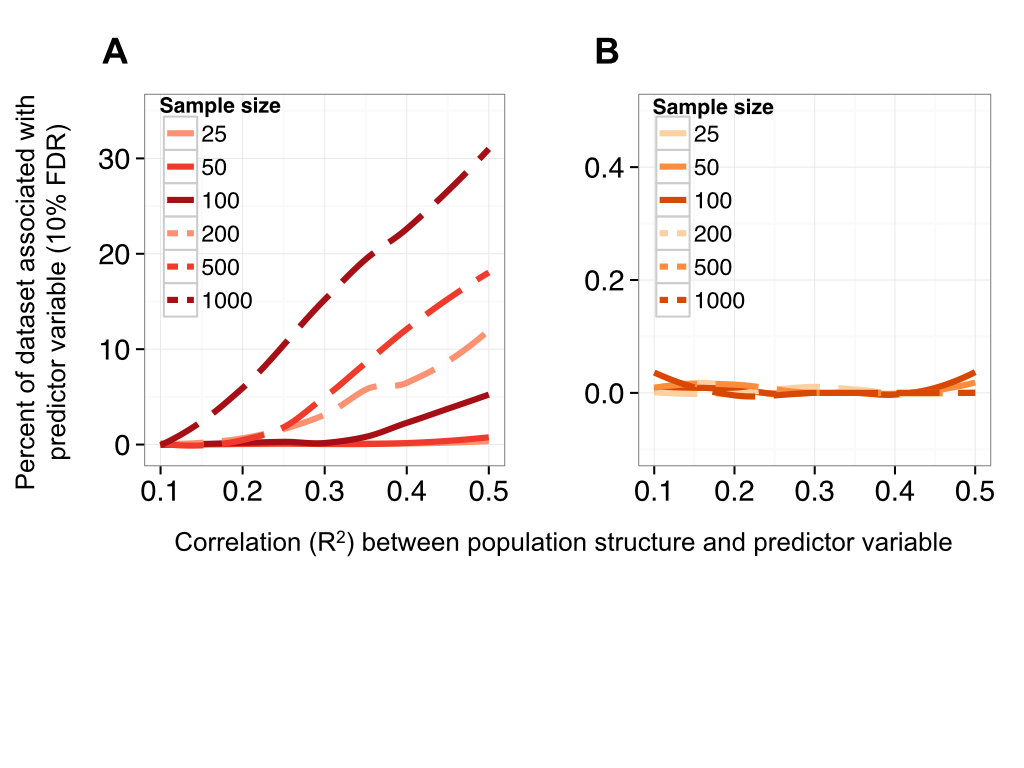

Supplement: S13 Fig — Percent of dataset associated with the predictor variable (at a 10% FDR) when simulated datasets (n = 5000 sites per dataset; h2 = 0.6) were analyzed with either (A) a beta-binomial model or (B) a binomial mixed model implemented in MACAU. The correlation between the simulated predictor variable and the first principal component of genome-wide genotype data is plotted on the x-axis. (TIFF) [file pgen.1005650.s013.tiff]

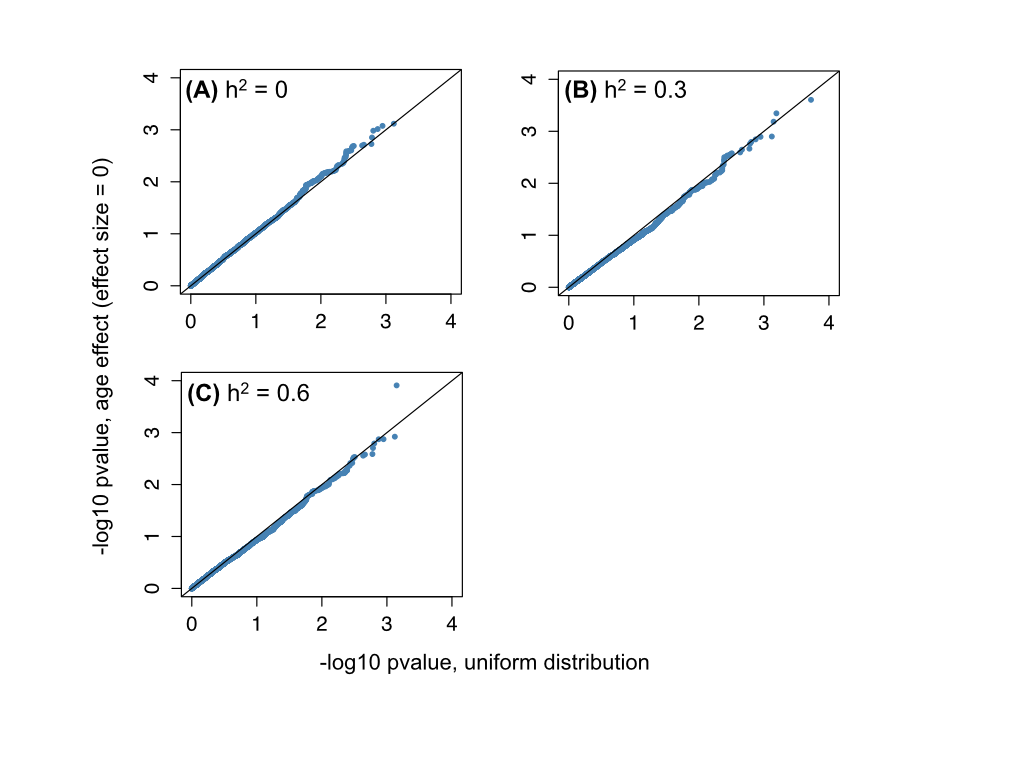

Supplement: S15 Fig — Results from 4500 simulated sites, where we set the effect of age on DNA methylation levels equal to 0 and the heritability of DNA methylation levels equal to (A) 0, (B) 0.3, or (C) 0.6. All QQ-plots compare the distribution of p-values produced by MACAU to the expected uniform distribution. (TIFF) [file pgen.1005650.s015.tiff]

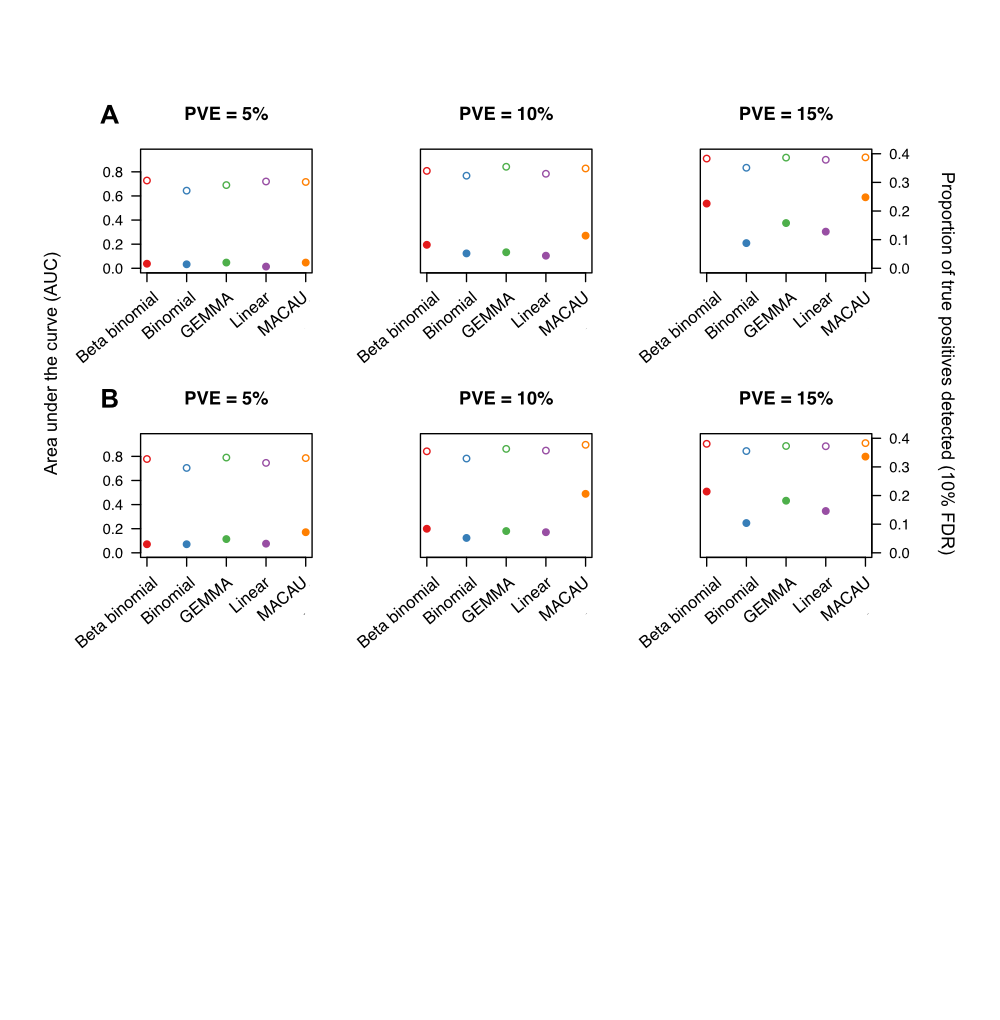

Supplement: S16 Fig — We simulated age effects on DNA methylation levels, in presence of genetic effects (panel A, h2 = 0.3; panel B, h2 = 0.6) across a range of effect sizes. The proportion of true positives detected at a 10% empirical FDR is plotted for each method (closed circles) as is the AUC (open circles). For all simulations shown here, the sample size was set to 80 individuals. (TIFF) [file pgen.1005650.s016.tiff]

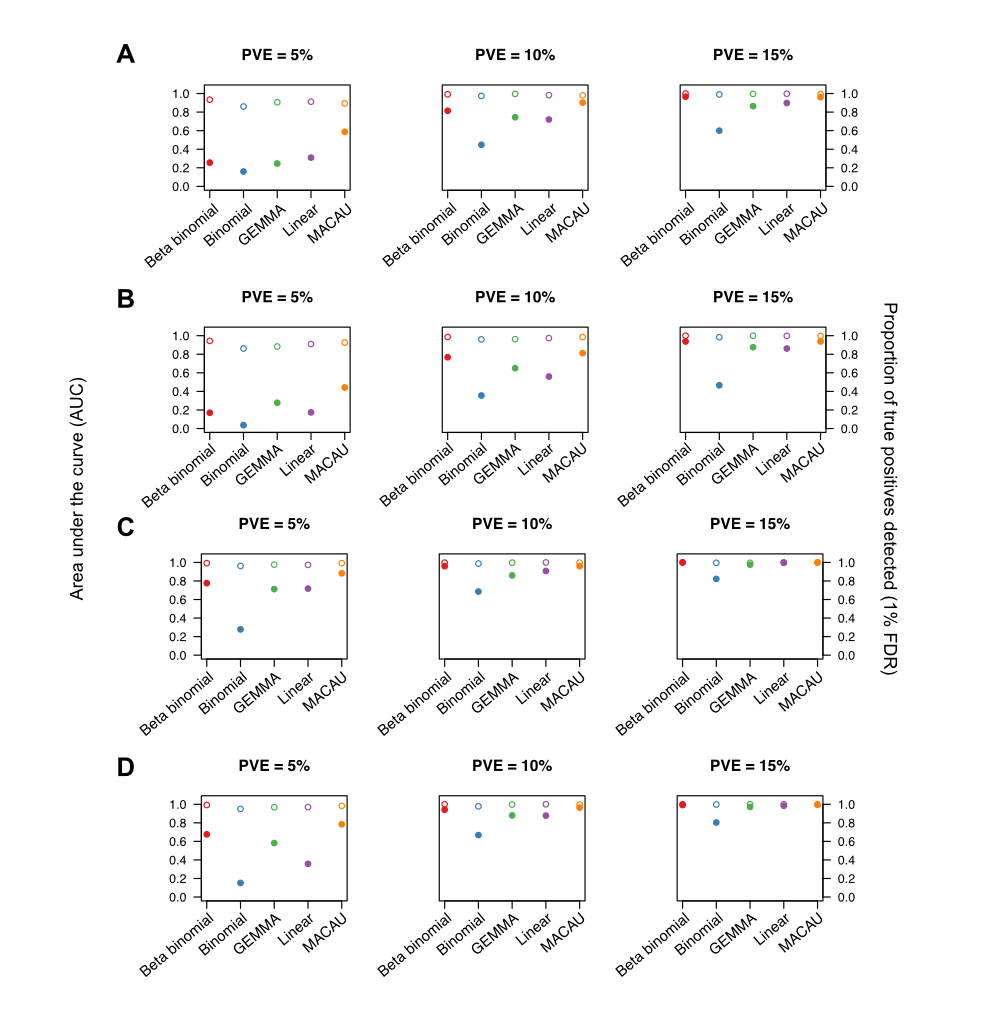

Supplement: S17 Fig — We simulated age effects on DNA methylation levels in datasets of 500 (A-B) and 1000 individuals (C-D). For all simulations, we included genetic effects on DNA methylation levels (panels A and C: h2 = 0.3; panels B and D: h2 = 0.6). Below, we show the proportion of true positives detected at a 1% empirical FDR (closed circles) as well as the AUC (open circles) for each method. (TIFF) [file pgen.1005650.s017.tiff]

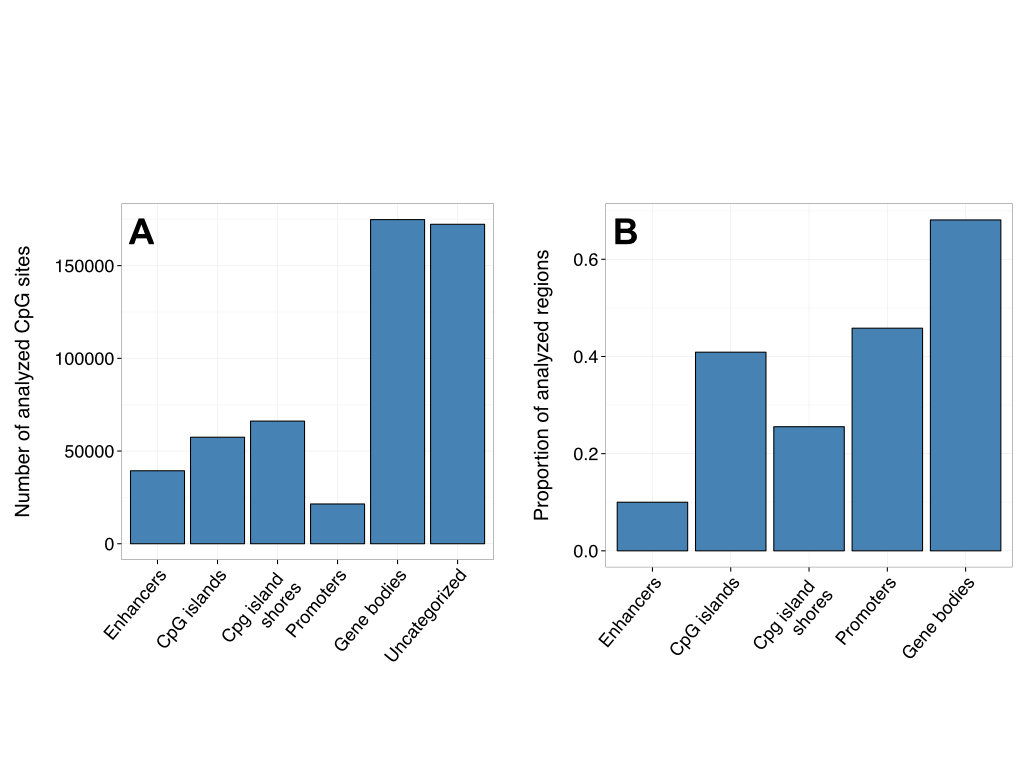

Supplement: S18 Fig — (A) Absolute number of sites analyzed for a given genomic region. See Materials and Methods for information on how we defined each genomic region. (B) Proportion of total annotated features in the baboon genome for which a least one CpG site was analyzed in this data set. (TIFF) [file pgen.1005650.s018.tiff]

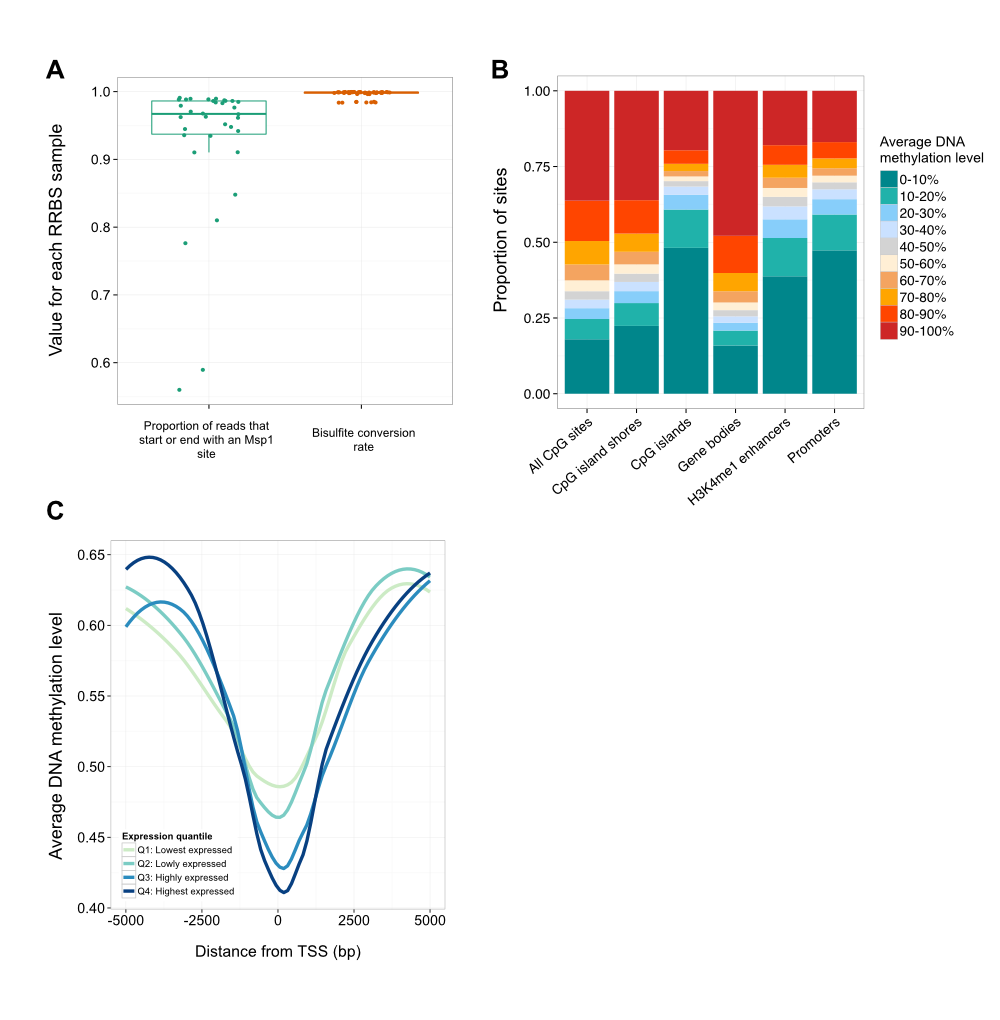

Supplement: S19 Fig — (A) The distributions of bisulfite conversion rates (estimated from a spike-in sample of unmethylated lambda phage DNA) and proportions of reads starting or ending with an Msp1 digest site, for each sample. (B) Barplots showing the distribution of DNA methylation levels by genomic compartment. As expected, CpG islands, H3K3me1-marked enhancers and promoters tend to be lowly methylated, while gene bodies and the background set of all sites analyzed tend to be hypermethylated. (C) For each CpG site within 5000 bp of an annotated Ensembl TSS, we calculated the mean DNA methylation level at that site across all 50 baboons. These mean levels are plotted as a smoothed function of distance from the TSS, stratified by gene expression level quartiles obtained from baboon whole blood RNA-seq. As expected, more highly methylated regions are associated with more lowly expressed genes. Only expressed genes were included. (TIFF) [file pgen.1005650.s019.tiff]

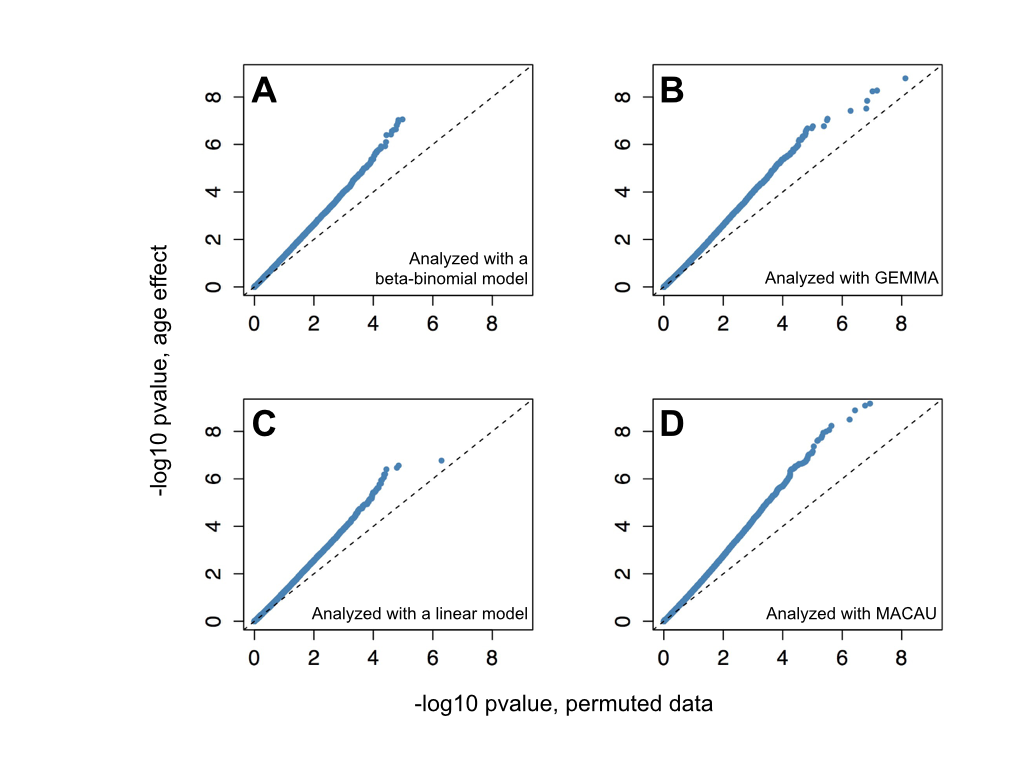

Supplement: S20 Fig — QQ-plots comparing the p-value distributions for (i) a model testing for effects of age on DNA methylation levels in real RRBS data, plotted on the y-axis; and (ii) the same model when the age values were permuted across individuals, plotted on the x-axis. For each method, the number of sites detected at a 10% FDR was as follows: Beta-binomial = 747, GEMMA = 205, Linear 324, MACAU = 1018. (TIFF) [file pgen.1005650.s020.tiff]

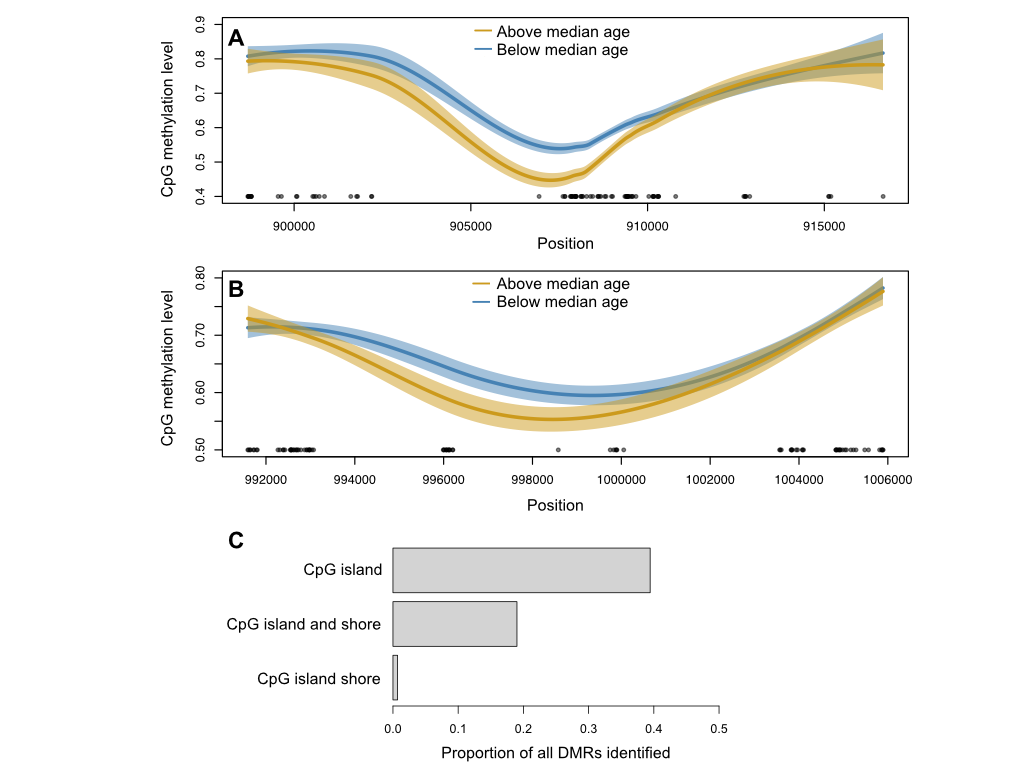

Supplement: S21 Fig — Overall, we detected 142 age-related DMRs. Two representative DMRs are plotted in panels A and B (location of DMR in panel A: Chr14, 908111–908168; and panel B: Chr 20: 996106–996139; see S5 Table for the locations of additional DMRs). To detect DMRs, baboon ages were binarized into two categories, based on whether an individual’s age fell above or below the median age in our sample. Smoothed estimates of DNA methylation levels are shown for each age group, and the location of measured CpG sites are noted along the x-axis by black dots. Panel C shows the proportion of all identified DMRs that fell in a CpG island, CpG island shore, or both. (TIFF) [file pgen.1005650.s021.tiff]

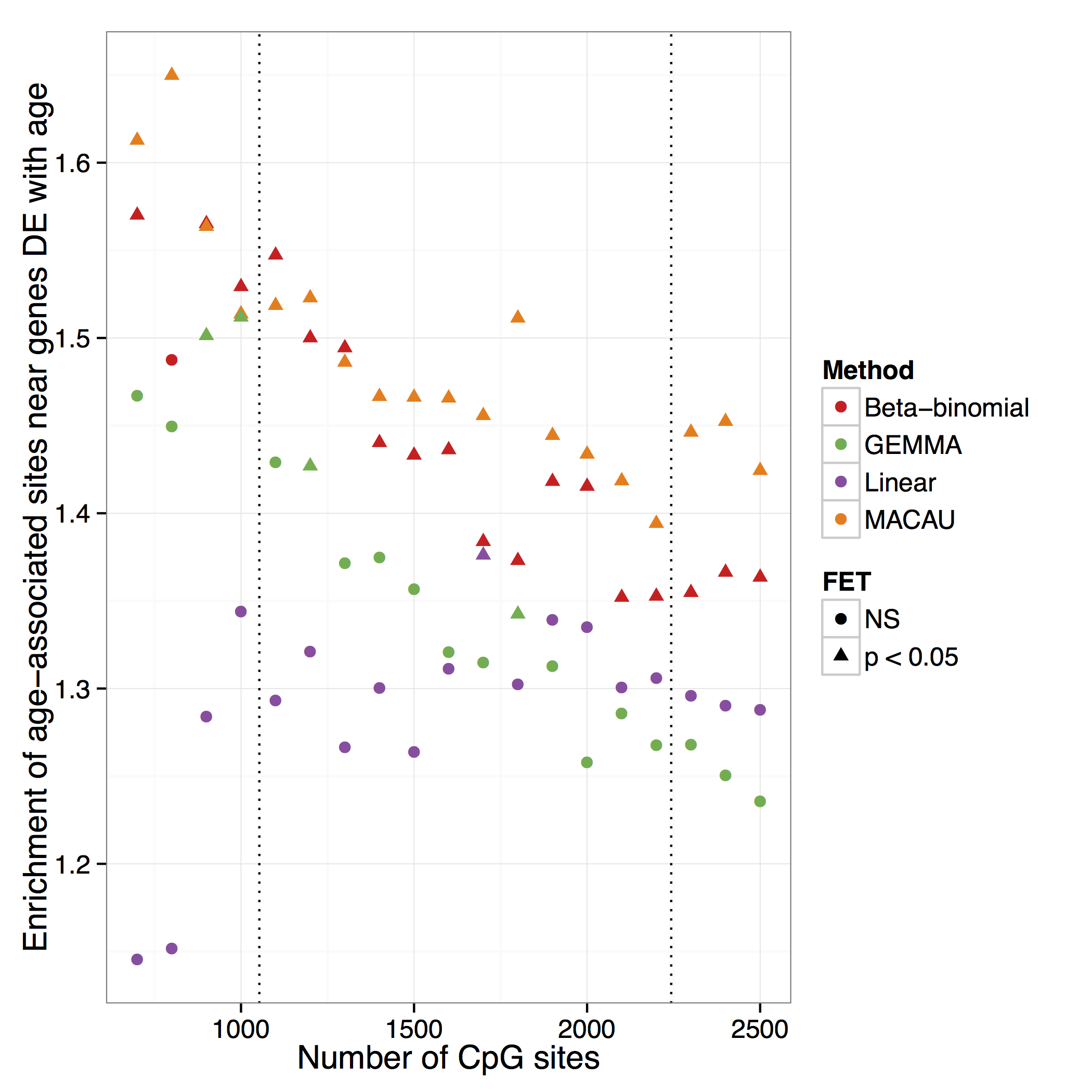

Supplement: S22 Fig — For each method below, we asked whether CpG sites that occur near age-associated genes were more likely to be differentially methylated with age compared to the background set of all CpG sites near genes (using a Fisher’s exact test). We report the enrichment observed and show whether the p-value associated with the Fisher’s exact test (FET) was below 0.05 (triangles). We repeated this analysis using a varying number of top CpG sites from each method, with the number for each analysis shown on the x-axis. Dotted vertical lines correspond to the number of sites detected by MACAU at a 10% empirical FDR (a more conservative approach), or at a 10% FDR calculated in the R package qvalue (a less conservative approach). (TIFF) [file pgen.1005650.s022.tiff]

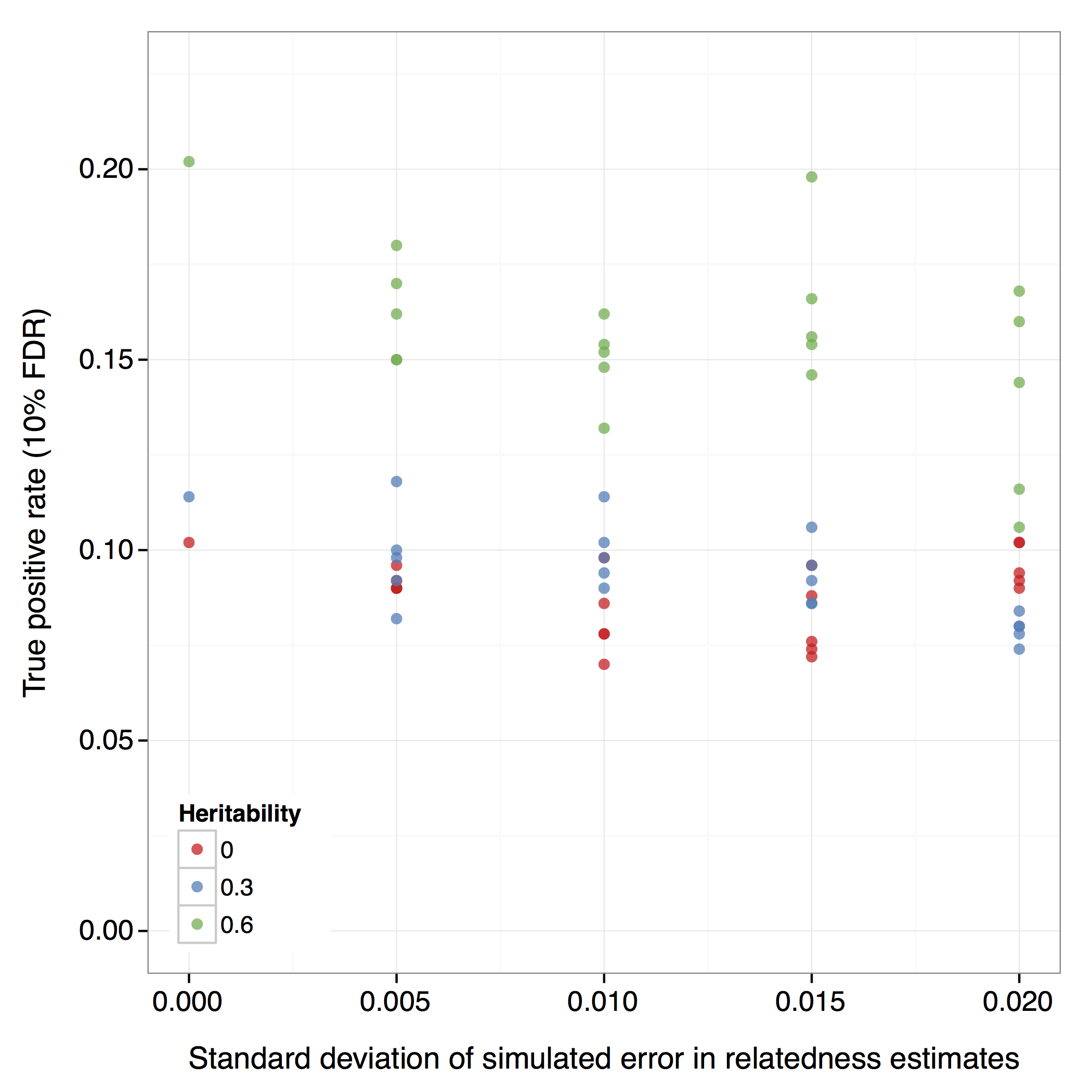

Supplement: S23 Fig — To understand how the performance of MACAU varies when there is error in the estimation of pairwise genetic relatedness, we added random error drawn from a normal distribution with mean 0 and standard error as noted on the x-axis. We then reran our analyses of all simulated datasets (with varying heritabilities, as noted in the Fig legend) where n = 80 and percent variance explained by age = 10%. For each analysis, we noted the number of simulated true positives detected by MACAU at a 10% empirical FDR (note, the results from our original analyses, with no error in the estimation of pairwise genetic relatedness, corresponds to x = 0). (TIFF) [file pgen.1005650.s023.tiff]

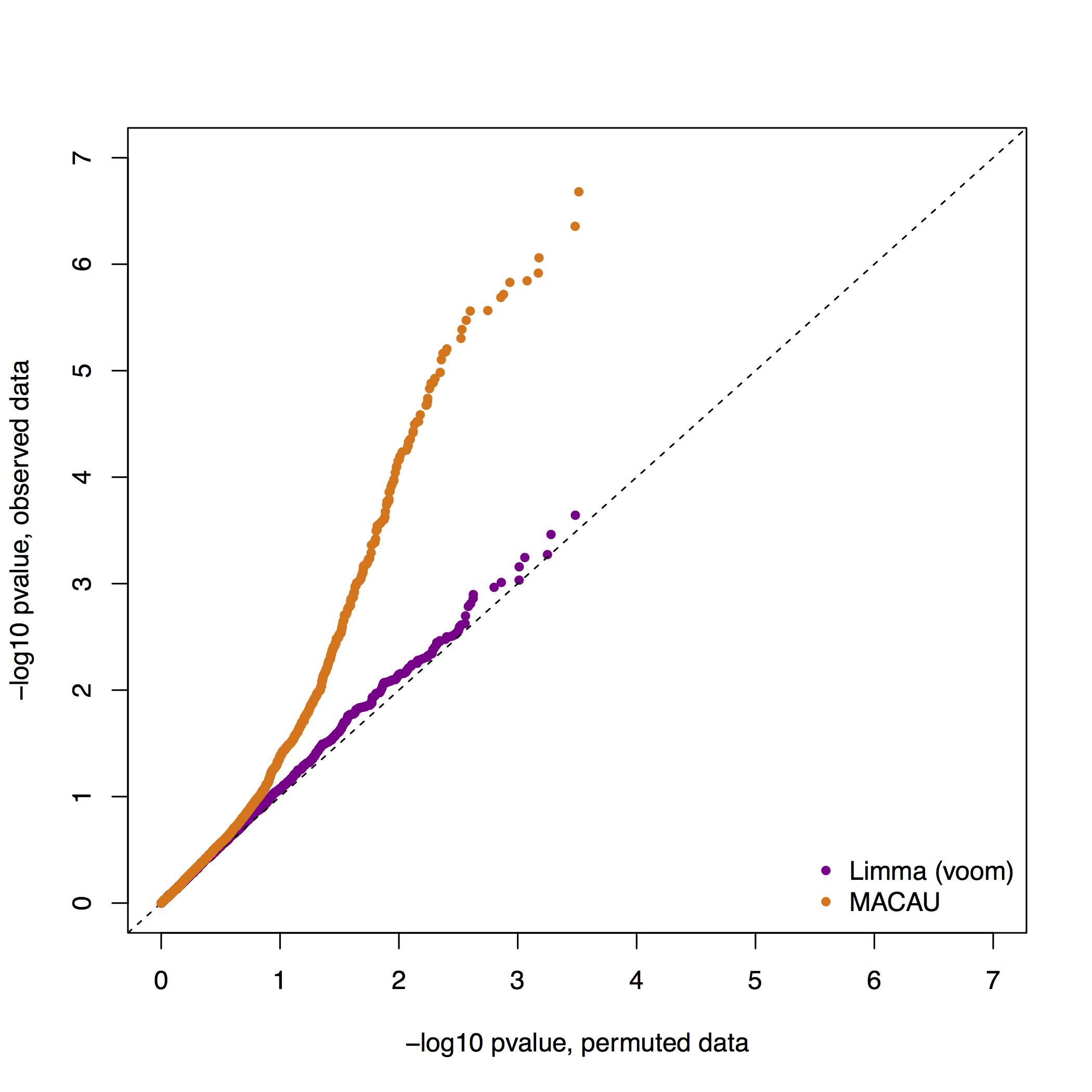

Supplement: S24 Fig — We tested the performance of a powerful, commonly used method for modeling RNA-seq data: the combination of the voom function for data weighting with limma, a linear model approach. To do so, we used simulated baboon bisulfite sequencing count data (n = 5000 sites including 500 true positives and 4500 true negatives; percent variance explained by age = 10%; sample size = 80, h2 = 0.6). QQ-plots show the results for both the voom + limma approach, as well as an analysis of the same dataset with MACAU. QQ-plots compare the p-value distributions for (i) a model testing for effects of age on DNA methylation levels, plotted on the y-axis; and (ii) the same model when the age values were permuted across individuals, plotted on the x-axis. MACAU detects 20.6% of simulated true positives (at a 10% empirical FDR), while the voom + limma approach detects less than 1% of simulated true positives. (TIFF) [file pgen.1005650.s024.tiff]
