# Supplementary material for: A Flexible, Efficient Binomial Mixed Model for Identifying Differential DNA Methylation in Bisulfite Sequencing Data
Source: PLoS Genet. 2015 Nov 24;11(11):e1005650. doi: 10.1371/journal.pgen.1005650 (PMC4657956; doi:10.1371/journal.pgen.1005650)
Supplement: S1 Table — A separate normal mixture distribution is used to approximate each negative log gamma distribution. The estimated parameters in the normal mixture distribution ensure that the Kullback-Leibler (KL) divergence between the two distributions is below 5x10^-4^. The parameters in the normal mixture distribution include the number of normal components (k), their weights (w), means (m) and variances (σ2). Means and variances are shown in their standardized version, where Ψ(r) denotes the diagamma function and Ψ’(r) denotes the trigamma function. (PDF) [file pgen.1005650.s025.pdf]

Table S1. Normal Mixture Approximations to -log(Ga(r, 1)) for r in [1, 5]. Normal mixture approximations to -log(Ga(r, 1)) for r in [1, 5]. A separate normal mixture distribution is used to approximate each negative log gamma distribution. The estimated parameters in the normal mixture distribution ensure that the Kullback-Leibler (KL) divergence between the two distributions is below $5x10^{-4}$. The parameters in the normal mixture distribution include the number of normal components (k), their weights (w), means (m) and variances (σ^2). Means and variances are shown in their standardized version, where Ψ(r) denotes the diagamma function and Ψ'(r) denotes the trigamma function.

		k									
r		1	2	3	4	5	6	7	8	9	10
1	w_{rk}	0.2924	0.2828	0.1625	0.09697	0.08053	0.05949	0.01882	0.005167	0.001194	0.0001863
	$\frac{m_{rk} + \Psi(r)}{\sqrt{\Psi'(r)}}$	-0.8141	0.02214	0.8407	-0.7554	-0.1850	1.753	2.747	3.857	5.168	6.964
	$\sigma_r^2 / \Psi'(r)$	0.1904									
2	w_{rk}	0.3960	0.3893	0.1640	0.04299	0.007059	0.0006391				
	$\frac{m_{rk} + \Psi(r)}{\sqrt{\Psi'(r)}}$	-0.7848	0.06357	1.023	2.126	3.460	5.249				
	$\sigma_r^2 / \Psi'(r)$	0.2892									
3	w_{rk}	0.4862	0.3882	0.1093	0.01527	0.001014					
	$\frac{m_{rk} + \Psi(r)}{\sqrt{\Psi'(r)}}$	-0.6673	0.3084	1.443	2.779	4.496					
	$\sigma_r^2 / \Psi'(r)$	0.3804									
4	w_{rk}	0.5861	0.3503	0.06010	0.003509						
	$\frac{m_{rk} + \Psi(r)}{\sqrt{\Psi'(r)}}$	-0.5422	0.5501	1.873	3.578						
	$\sigma_r^2 / \Psi'(r)$	0.4660									
5	w_{rk}	0.5617	0.3696	0.06518	0.003533						
	$\frac{m_{rk} + \Psi(r)}{\sqrt{\Psi'(r)}}$	-0.5582	0.5013	1.784	3.439						
	$\sigma_r^2 / \Psi'(r)$	0.4829									
