# Supplementary material for: A Flexible, Efficient Binomial Mixed Model for Identifying Differential DNA Methylation in Bisulfite Sequencing Data
Source: PLoS Genet. 2015 Nov 24;11(11):e1005650. doi: 10.1371/journal.pgen.1005650 (PMC4657956; doi:10.1371/journal.pgen.1005650)
Supplement: S1 Text — (PDF) [file pgen.1005650.s030.pdf]

Text S1: Detailed Methods

1 Binomial Mixed Model

To detect differentially methylated sites, we model each potential target of DNA methylation one site at a time. For each site, we consider the following binomial mixed model (BMM):

$$y_i \sim \text{Bin}(r_i, \pi_i), \tag{1}$$

where r_i is the total read count for ith individual; y_i is the methylated read count for that individual, constrained to be an integer value less than or equal to r_i; and π_i is an unknown parameter that represents the true proportion of methylated reads for the individual at the site. We use a logit link to model π_i as a linear function of parameters:

$$\text{logit}(\pi_i) = \log(\lambda_i) = \mathbf{w}_i^T \boldsymbol{\alpha} + x_i\beta + g_i + e_i, \tag{2}$$

$$\mathbf{g} = c(g_1, \cdots, g_n)^T \sim \text{MVN}(0, \sigma^2 h^2 \mathbf{K}), \tag{3}$$

$$\mathbf{e} = c(e_1, \cdots, e_n)^T \sim \text{MVN}(0, \sigma^2(1-h^2)\mathbf{I}_{n\times n}), \tag{4}$$

where logit denotes a logistic transformation $\text{logit}(\pi_i) = \log(\frac{\pi_i}{1-\pi_i})$; $\lambda_i = \frac{\pi_i}{1-\pi_i}$ is the odds; $\mathbf{w}_i$ is a c-vector of covariates including an intercept and $\boldsymbol{\alpha}$ is a c-vector of corresponding coefficients; x_i is the predictor of interest and β is its coefficient; $\mathbf{g}$ is an n-vector of genetic random effects that model correlation due to population structure or individual relatedness; $\mathbf{e}$ is an n-vector of environmental residual errors that model independent variation; $\mathbf{K}$ is a known n by n relatedness matrix that can be calculated based on a pedigree or genotype data and that has been standardized to ensure $tr(\mathbf{K})/n = 1$ (this ensures that h^2 lies between 0 and 1, and can be interpreted as heritability, see [1]); $\mathbf{I}$ is an n by n identity matrix; $\sigma^2 h^2$ is the genetic variance component; $\sigma^2(1-h^2)$ is the environmental variance component; h^2 is the heritability of the logit transformed methylation proportion (i.e. $\text{logit}(\pi)$); and MVN denotes the multivariate normal distribution.

The binomial mixed model proposed here belongs to the generalized linear mixed model family [2]. Both $\mathbf{g}$ and $\mathbf{e}$ model over-dispersion, the increased variance in the data that is not explained by the binomial model. However, they model different aspects of over-dispersion: $\mathbf{e}$ models the variation that is due to independent environmental noise (a known problem in data sets based on sequencing reads), while $\mathbf{g}$ models the variation that is explained by kinship or population structure. Effectively, our model improves and generalizes the previous beta binomial model by introducing this extra $\mathbf{g}$ term to model individual relatedness due to kinship, population structure, or stratification.

2 Inference Method Overview

We are interested in testing the null hypothesis $H_0 : \beta = 0$. This requires obtaining the maximum likelihood estimate $\hat{\beta}$ from the model. Unlike its linear counter-part, obtaining the estimate of β from the binomial mixed model is not a trivial task, as the joint likelihood consists of an n-dimensional integral that cannot be solved analytically [2]. Previous frequentist approaches to address this problem include direct numerical integration using Gauss-Hermite quadrature [3], or Laplace approximation that is applied to the likelihood function [4] or the quasi-likelihood function [5–8]. However, both numerical integration and analytic approximation do not scale well with the increasing dimension of the integral, which unfortunately equals the sample size in our model. Even a second order Laplace approximation yields a biased estimate and overly narrow confidence interval, especially when the uncertainty in the variance component estimate is large [9–13]. Therefore, frequentist approaches for estimation and inference in the binomial mixed model remain notoriously difficult and is still an active area of research [14].

In contrast to the frequentist methods, Markov chain Monte Carlo (MCMC)-based Bayesian approaches provide an appearing alternative [11]. Bayesian methods naturally account for the uncertainty in the variance component estimates and can achieve arbitrary inference accuracy if the chain is allowed to run long enough. Despite these attractive theoretical features, however, constructing an efficient MCMC algorithm for practical problems is not easy. Previous MCMC approaches for generalized linear mixed models either require a normal approximation to the likelihood function that diminishes its gains over the frequentist methods [15,16], or use n-steps of Metropolis–Hastings algorithm to sample the n-dimensional latent rate variables where efficient proposal distributions for all of them can be hard to construct [17,18]. To improve upon these previous approaches, a new MCMC algorithm [19–21] has been recently developed based on auxiliary variable representation of the binomial distribution [22]. By introducing latent variables to replace the observed count data, the algorithm makes sampling and computation relatively straightforward.

Therefore, we rely on this particular form of MCMC in the present study. Our main contribution is to further develop an accurate approximation to the distribution of these latent variables, where the approximation form is specifically designed to allow us to adapt recent mixed model innovations [23–26] that substantially reduce the computational burden. By using a mean-normal mixture approximation to the negative log gamma distribution, our approach reduces the per-MCMC iteration computational complexity from $O(n^3)$ to $O(n^2)$, where n is the sample size. This modification allows the binomial mixed model to be efficiently applied to hundreds of individuals and millions of methylation sites.

Although we use MCMC for posterior sampling, our primary goal is not to perform a Bayesian analysis by producing Bayes factors for model comparison (although this is an interesting area to explore in the future). Rather, our goal is to use MCMC as a convenient and accurate tool to obtain the marginal likelihood of β that is otherwise infeasible or inaccurate to obtain under various frequentist approaches. Under asymptotics, both the likelihood function and the marginal posterior distribution for β will be approximately normal [27]. Since the likelihood function is simply the difference between the posterior and the prior, once we have obtained the posterior mean and standard deviation of β and paired these values to their prior counter-parts, we can easily obtain the approximate likelihood function and compute the approximate maximum likelihood estimate $\hat{\beta}$ and its standard error $se(\hat{\beta})$ using the method of moments. We can then construct approximate Wald test statistics and p values for hypothesis testing.

In the present study, we use flat priors for all nuisance parameters $(\boldsymbol{\alpha}, \sigma^2, h^2)$, or $p(\boldsymbol{\alpha}) \propto 1$, $p(\sigma^2) \propto 1$ and $p(h^2) \propto 1$. (Notice that a uniform distribution for σ^2 on the log scale, or $p(\log(\sigma^2)) \propto 1$, would make the posterior distribution different from the likelihood.) For the parameter of interest, β, we could also use a flat prior, in which case the posterior would be the likelihood. For computational stability reasons, however, we use a relatively informative prior, $\beta \sim N(0, \sigma_b^2)$ instead. A relatively informative prior restricts the sampling space when the likelihood is not informative, allowing efficient posterior sampling. Since we rely on the difference betwen the posterior and the prior for approximate inference, the choice of prior for β does not influence the eventual results. In the present study, we set $\sigma_b^2 = 1$.

Applications to real data confirm that this procedure produces well-calibrated p-values (Figure 1), suggesting that a few dozen samples are large enough to ensure asymptotic behavior. Moreover, although our approach is inherently stochastic – because the posterior mean and standard deviation of β may be slightly different for different chains – we show that a thousand MCMC iterations per site is large enough to produce stable estimates of the test statistics and p values (Figure S2).

3 The MACAU Algorithm

Below, we describe the MACAU algorithm, for Mixed model Association for Count data via data AUgmentation, in detail.

3.1 Data Augmentation

To bypass the difficult likelihood function that results from the count nature of the data, we introduce continuous auxiliary variables to replace y_i. For ith individual, observing y_i methylated reads out of r_i total reads is equivalent to observing a sequence of r_i binary read indicators $(y_{i1}, \cdots, y_{ir_i})$, where $y_{ij} = 1$ indicates that the jth read is a methylated read and $y_{ij} = 0$ indicates otherwise. Obviously, $y_i = \sum_{j=1}^{r_i} y_{ij}$. We can view each y_{ij} as a random variable generated from a logistic regression model with mean $\log(\lambda_i)$. We further introduce a continuous latent variable u_{ij} [19, 20], often referred to as a utility [22], such that

$$u_{ij} = \log(\lambda_i) + \epsilon_{ij}^1, \quad \epsilon_{ij}^1 \sim \text{EV}(0, 1), \tag{5}$$

where EV(0, 1) denotes a standard type-1 extreme value distribution with density function $e^{-x}e^{-e^{-x}}$. Then

$$y_{ij} = \begin{cases} 1, & \text{if } u_{ij} > \epsilon_{ij}^0, \\ 0, & \text{otherwise}, \end{cases} \tag{6}$$

where $\epsilon_{ij}^0 \sim \text{EV}(0, 1)$. The above two equations come from the fact that the difference between two type-1 extreme value distributed random variables follows a logistic distribution, and a random variable that follows a logistic distribution serves as a liability variable for a logistic regression [22].

The attractive feature of introducing this set of independently and identically distributed u_{ij} is that, conditional on all u_{ij}, the posterior of $(\boldsymbol{\alpha}, \beta, \sigma^2, h^2)$ no longer depends on the observed methylated read indicator y_{ij}, hence removing the non-linearity constraint that comes with the binomial aspect of our model. Applying the relationship between the EV distribution and the exponential distribution, we have $e^{-u_{ij}} \sim \text{Exp}(\lambda_i)$ and $e^{-\epsilon_{ij}^0} \sim \text{Exp}(1)$, where Exp denotes the exponential distribution. This relationship allows us to easily sample u_{ij} conditional on λ_i and y_{ij} based on the convenient exponential distribution rather than the more difficult EV distribution, as $e^{-u_{ij}} \sim \text{Exp}(1 + \lambda_i)$ if $y_{ij} = 1$ and $e^{-u_{ij}} \sim \text{Exp}(1 + \lambda_i) + \text{Exp}(\lambda_i)$ if $y_{ij} = 0$.

An undesirable feature of the above approach, however, is that we have to work with a much larger latent space of u_{ij} than the original n observations of y_i. Effectively, we have to retain the data at the individual read level. This drawback can be mitigated by combining all exponentiated negative latent utilities together [21], by introducing a new latent variable

$$z_i = -\log(\sum_{j=1}^{r_i} e^{-u_{ij}}) = \log(\lambda_i) + \epsilon_i, \tag{7}$$

where $\epsilon_i = -\log(\sum_{j=1}^{r_i} e^{-\epsilon_{ij}^1})$ follows a negative log gamma distribution, $-\log(\text{Ga}(r_i, 1))$; Ga denotes a gamma distribution with the two parameters representing shape and rate, respectively. This is because a gamma random variable is a summation of independent exponential random variables with a same rate parameter.

Using the latent variable z_i instead of u_{ij} reduces the size of the latent space back to the observed space. Conditional on z_i, we again do not need to use y_i, allowing us to bypass the count feature of the observed data in the algorithm.

3.2 Normal Mixture Approximation

To further circumvent the difficulty introduced by the non-normality of ϵ_i, we follow previous ideas [20, 21] to approximate the non-normal distribution by using a mixture of normals. Importantly, we take advantage of recent innovations in efficient mixed model algorithms [23–26] by using a mean mixture of normals where each normal distribution has a different mean but share the same variance.

Specifically, for every possible integer value of r, we identify a normal approximation in the form of $\sum_{k=1}^{k_r} w_{rk}\text{N}(m_{rk}, s_r^2)$, to the negative log gamma distribution $-\log(\text{Ga}(r, 1))$. Because the mean $(-\Psi(r)$,

where Ψ denotes a digamma function) and the variance ($\Psi'(r)$, where Ψ' denotes a trigamma function) of the negative log gamma distribution is a function of r, to ensure approximation stability we work on the standardized version of the negative log gamma distribution, by centering with the mean and standardizing with the standard deviation. Then, we estimate the number of components k_r, the weights w_{rk}, the means m_{rk} and the variance s_r^2 via the Nelder-Mead algorithm by minimizing the Kullback–Leibler (KL) divergence between the two distributions. These parameter estimates ensure that the KL divergence is smaller than 0.0005, so that the difference between the approximate and the exact distributions are ignorable in practice. Because the negative log gamma distribution asymptotically approximates a normal distribution, the approximation becomes easier for larger r. Therefore, we can use increasingly smaller number of normal components for accurate approximation.

For small values of r ($r \in [1, 5]$), we provide detailed parameter values in Table S1. For median values of r ($r \in [6, 169]$), we no longer need to store parameters for every r. Instead, we can interpolate the weight, mean and variance estimates across the range of r using rational functions without loss of accuracy. These functions are provided in the Table S2. For large values of r ($r \in [170, \infty)$, we use a single normal distribution $\mathrm{N}(0, \Psi'(r))$ for approximation. The mean normal mixture approximations are accurate. Even in the most difficult case where $r = 1$, we only observe small difference between the approximate and the exact distributions (Figure S3).

3.3 Detailed Sampling Steps and Efficient Computation

Now we are ready to describe the detailed MCMC algorithm. Here, with the normal mixture approximation, we have

$$z_i = \log(\lambda_i) + \epsilon_i = \mathbf{w}_i^T \boldsymbol{\alpha} + x_i\beta_i + g_i + e_i + \epsilon_i, \quad \epsilon_i \sim \sum_{k=1}^{k_{r_i}} w_{r_i k} \mathrm{N}(m_{r_i k}, s_{r_i}^2). \tag{8}$$

We introduce a vector of latent indicators $\boldsymbol{\gamma} = (\gamma_1, \cdots, \gamma_n)$, where each $\gamma_i \in (1, \cdots, k_{r_i})$ indicates which normal component the corresponding ϵ_i is from. Conditional on z_i and $(\boldsymbol{\alpha}, \beta, g_i, e_i)$, we have

$$P(\gamma_i = k | z_i, \boldsymbol{\alpha}, \beta, g_i, e_i) \propto w_{r_i k} \Phi(z_i - \log(\lambda_i) - m_{r_i k}, \sigma_{r_i}^2), \tag{9}$$

where $k \in (1, \cdots, k_{r_i})$ and Φ denotes the normal density function. Conditional on $\boldsymbol{\gamma}$, we can integrate out $\boldsymbol{\alpha}$, β, $\mathbf{g}$, $\mathbf{e}$ and $\boldsymbol{\epsilon}$ analytically to obtain the marginal distribution of σ^2 and h^2,

$$P(\sigma^2, h^2 | \mathbf{z}, \boldsymbol{\gamma}) \propto |\mathbf{H}|^{-\frac{1}{2}} |\mathbf{W}^T \mathbf{H}^{-1} \mathbf{W}|^{-\frac{1}{2}} |\sigma_b^2 \mathbf{x}^T \mathbf{P}_w \mathbf{x} + 1|^{-\frac{1}{2}} e^{-\frac{1}{2}(\mathbf{z}-\mathbf{m}_{\boldsymbol{\gamma}})^T \mathbf{P}_x (\mathbf{z}-\mathbf{m}_{\boldsymbol{\gamma}})}, \tag{10}$$

where $\mathbf{z} = (z_1, \cdots, z_n)^T$, $\mathbf{m}_{\boldsymbol{\gamma}} = (m_{r_1\gamma_1}, \cdots, m_{r_n\gamma_n})^T$, $\mathbf{W} = (\mathbf{w}_1, \cdots, \mathbf{w}_n)^T$, $\mathbf{D}_\mathbf{r}$ is an n by n diagonal matrix with iith element $\sigma_{r_i}^2$, $\mathbf{V} = h^2\mathbf{K} + (1-h^2)\mathbf{I}$, $\mathbf{H} = \sigma^2\mathbf{V} + \mathbf{D}_\mathbf{r}$, $\mathbf{P}_w = \mathbf{H}^{-1} - \mathbf{H}^{-1}\mathbf{W}^T(\mathbf{W}^T\mathbf{H}^{-1}\mathbf{W})^{-1}\mathbf{W}\mathbf{H}^{-1}$ and $\mathbf{P}_x = \mathbf{P}_w - \mathbf{P}_w\mathbf{x}(\mathbf{x}^T\mathbf{P}_w\mathbf{x} + \sigma_b^{-2})^{-1}\mathbf{x}^T\mathbf{P}_w$.

We can use the Metropolis–Hastings (MH) algorithm to obtain posterior samples for σ^2 and h^2 jointly. Afterwards, we can obtain posterior samples for $\boldsymbol{\alpha}$, β and $\mathbf{g} + \mathbf{e}$ in turn,

$$P(\beta | \mathbf{z}, \boldsymbol{\gamma}, \sigma_g^2, \sigma_e^2) \sim \mathrm{N}((\mathbf{x}^T\mathbf{P}_w\mathbf{x} + \sigma_b^{-2})^{-1}\mathbf{x}^T\mathbf{P}_w(\mathbf{z} - \mathbf{m}_{\boldsymbol{\gamma}}), (\mathbf{x}^T\mathbf{P}_w\mathbf{x} + \sigma_b^{-2})^{-1}), \tag{11}$$

$$P(\boldsymbol{\alpha} | \mathbf{z}, \boldsymbol{\gamma}, \beta, \sigma_g^2, \sigma_e^2) \sim \mathrm{MVN}((\mathbf{W}^T\mathbf{H}^{-1}\mathbf{W})^{-1}\mathbf{W}^T\mathbf{H}^{-1}(\mathbf{z} - \mathbf{m}_{\boldsymbol{\gamma}} - \mathbf{x}\beta), (\mathbf{W}^T\mathbf{H}^{-1}\mathbf{W})^{-1}), \tag{12}$$

$$P(\mathbf{g} + \mathbf{e} | \mathbf{z}, \boldsymbol{\gamma}, \boldsymbol{\alpha}, \beta, \sigma^2, h^2) \sim \mathrm{MVN}(\sigma^2\mathbf{V}\mathbf{H}^{-1}(\mathbf{z} - \mathbf{m}_{\boldsymbol{\gamma}} - \mathbf{W}\boldsymbol{\alpha} - \mathbf{x}\beta), \sigma^2\mathbf{V}\mathbf{H}^{-1}\mathbf{D}_\mathbf{r}). \tag{13}$$

Finally, conditional on y_i and λ_i, the posterior of z_i is easy to sample. By using the relationship between the gamma distribution and the exponential distribution, we have

$$z_i | y_i, \lambda_i \sim \mathrm{Ga}(r_i, 1 + \lambda_i) + \mathrm{Ga}(y_i, \lambda_i). \tag{14}$$

The most computationally expensive part of the algorithm is the MH step: a naive approach to evaluate $P(\sigma^2, h^2|z_i, \gamma_i)$ would involve cubic operations. Our mean normal mixture approximation allows us to evaluate this marginal likelihood efficiently as we can apply here the mixed model innovations developed recently [23–26]. This is because given the observed data, $\mathbf{D_r}$ is a fixed diagonal matrix where the elements do not depend on a $\boldsymbol{\gamma}$ that changes in every MCMC iteration. Therefore, for a given matrix $\mathbf{V}$, we can perform an eigen-decomposition on $\mathbf{D_r}^{-\frac{1}{2}}\mathbf{V}\mathbf{D_r}^{-\frac{1}{2}} = \mathbf{U}\mathbf{D}\mathbf{U}^T$. This allows us to decompose $\mathbf{H} = \sigma^2\mathbf{V} + \mathbf{D_r} = \mathbf{D_r}^{\frac{1}{2}}\mathbf{U}(\sigma^2\mathbf{D} + \mathbf{I})\mathbf{U}^T\mathbf{D_r}^{\frac{1}{2}}$. Afterwards, we can transform the latent variables and other covariates to obtain $\mathbf{D_r}^{\frac{1}{2}}\mathbf{U}(\mathbf{z} - \mathbf{m}_{\boldsymbol{\gamma}})$, $\mathbf{D_r}^{\frac{1}{2}}\mathbf{U}\mathbf{W}$ and $\mathbf{D_r}^{\frac{1}{2}}\mathbf{U}\mathbf{x}$. This procedure avoids any cubic operations later on in the MCMC steps. Therefore, with the mean normal mixture approximation, we only need to perform eigen-decompositions at the beginning of the MCMC. Afterwards, each Gibbs step only requires quadratic operations (transformation of $\mathbf{z} - \mathbf{m}_{\boldsymbol{\gamma}}$). In practice, because $\mathbf{V}$ is a function of h^2, we assign a discrete uniform prior for h^2 and evaluate the eigen-decompositions on every discrete values of h^2. In the present study, we found that using either 10 or 100 discrete values of h^2 yields almost identical results (and we present the analyses results for the formal in the main text), suggesting that a fine grid for h^2 is not necessary because of our small sample size. Finally, for all analyses in the present study, we ran 1100 Gibbs sampling iterations with the first 100 as burn-in. In each Gibbs iteration, after sampling the latent variables $\mathbf{z}$ and the latent indicators $\boldsymbol{\gamma}$, we further ran 10 MH steps before continuing the Gibbs iterations.

4 Parameter Estimation and p Value Computation

Denote $\bar{\beta}$ as the posterior mean and σ_β^2 as the posterior variance. Since both the likelihood and the posterior follow normal distributions asymptotically, and because we also use a normal distribution as the prior distribution, we can easily obtain the approximate maximum likelihood estimate and its standard error by the method of moments, or

$$\hat{\beta} = \sigma_b^2\bar{\beta}/(\sigma_b^2 - \sigma_\beta^2), \tag{15}$$

$$se(\hat{\beta}) = \sigma_b\sigma_\beta/\sqrt{\sigma_b^2 - \sigma_\beta^2}. \tag{16}$$

The condition $\sigma_b^2 > \sigma_\beta^2$ is guaranteed by asymptotics. In rare cases, however, this condition may not be satisfied because of the limited MCMC sampling iterations in practice. This may be particularly concerning for sites where the likelihood function is not informative. Arguably, these non-informative sites are the ones that we do not want to perform analysis on in the first place. Therefore, this condition gives us a natural way to perform post-filtering. In the software implementation, we do not analyze sites where $\sigma_\beta^2 \geq c\sigma_b^2$ for a user defined threshold c ($c \leq 1$). We use $c = 0.95$ throughout the present study. This post-filtering step, however, has minimal influence on the results, as only a few dozen sites, out of half a million, are filtered out in each analysis.

References

1. Zhou X, Carbonetto P, Stephens M (2013) Polygenic modelling with Bayesian sparse linear mixed models. PLoS Genetics 9: e1003264.

2. McCulloch CE, Searle SR, Neuhaus JM (2008) Generalized, Linear, and Mixed Models. New York, NY, USA: Wiley-Interscience.

3. Pinheiro JC, Chao EC (2006) Efficient Laplacian and adaptive Gaussian quadrature algorithms for multilevel generalized linear mixed models. Journal of Computational and Graphical Statistics 15: 58-81.

4. Raudenbush SW, Yang ML, Yosef M (2000) Maximum likelihood for generalized linear models with nested random effects via high-order, multivariate Laplace approximation. Journal of Computational and Graphical Statistics 9: 141-157.

5. Goldstein H (1991) Nonlinear multilevel models with an application to discrete response data. Biometrika 78: 45-51.

6. Breslow NE, Clayton DG (1993) Approximate inference in generalized linear mixed models. Journal of the American Statistical Association 88: 9-25.

7. Breslow NE, Lin X (1995) Bias correction in generalised linear mixed models with a single component of dispersion. Biometrika 82: 81-91.

8. Lin X, Breslow NE (1996) Bias correction in generalised linear mixed models with multiple components of dispersion. Journal of the American Statistical Association 91: 1007-1016.

9. Goldstein H, Rasbash J (1996) Improved approximations for multilevel models with binary responses. Journal of the Royal Statistical Society Series A 159: 505-513.

10. Rodriguez G, Goldman N (2001) Improved estimation procedures for multilevel models with binary response: A case-study. Journal of the Royal Statistical Society Series A 164: 339-355.

11. Browne WJ, Draper D (2006) A comparison of Bayesian and likelihood-based methods for fitting multilevel models. Bayesian Analysis 3: 473-514.

12. Jang W, Lim J (2009) A numerical study of PQL estimation biases in generalized linear mixed models under heterogeneity of random effects. Communications in Statistics - Simulation and Computation 38: 692-702.

13. Fong Y, Rue H, Wakefield J (2010) Bayesian inference for generalized linear mixed models. Biostatistics 11: 397-412.

14. Tuerlinckx F, Rijmen F, Verbeke G, Boeck PD (2006) Statistical inference in generalized linear mixed models: A review. British Journal of Mathematical and Statistical Psychology 59: 225-255.

15. Zeger SL, Karim MR (1991) Generalized linear models with random effects: A Gibbs sampling approach. Journal of the American Statistical Association 86: 79-86.

16. Karim MR, Zeger SL (1992) Generalized linear models with random effects: salamander mating revisited. Biometrics 48: 631-644.

17. Clayton DG (1996) Generalized linear mixed models. In: Gilks, W. R., Richardson, S. and Spiegelhalter, D. J. (editors), Markov Chain Monte Carlo in Practice. London, UK: Chapman and Hall.

18. Gamerman D (1997) Sampling from the posterior distribution in generalized linear mixed models. Statistics and Computing 7: 57-68.

19. Scott SL (2011) Data augmentation, frequentistic estimation, and the Bayesian analysis of multinomial logit models. Statistical Papers 52: 87-109.

20. Fruhwirth-Schnatter S, Fruhwirth R (2007) Auxiliary mixture sampling with applications to logistic models. Computational Statistics and Data Analysis 51: 3509-3528.

21. Fruhwirth-Schnatter S, Fruhwirth R, Held L, Rue H (2009) Improved auxiliary mixture sampling for hierarchical models of non-Gaussian data. Statistics and Computing 19: 479-492.

22. McFadden D (1974) Conditional logit analysis of qualitative choice behaviour. In: Zarembka, P. (Ed.), Frontiers of Econometrics. New York, NY, USA: Academic Press.

23. Kang HM, Zaitlen NA, Wade CM, Kirby A, Heckerman D, et al. (2008) Efficient control of population structure in model organism association mapping. Genetics 178: 1709-1723.

24. Lippert C, Listgarten J, Liu Y, Kadie CM, Davidson RI, et al. (2011) FaST linear mixed models for genome-wide association studies. Nature Methods 8: 833-835.

25. Zhou X, Stephens M (2012) Genome-wide efficient mixed-model analysis for association studies. Nature Genetics 44: 821-824.

26. Pirinen M, Donnelly P, Spencer CCA (2013) Efficient computation with a linear mixed model on large-scale data sets with applications to genetic studies. Annals of Applied Statistics 7: 369-390.

27. Schwartz L (1965) On Bayes procedures. Zeitschrift fur Wahrscheinlichkeitstheorie und Verwandte Gebiete 4: 10-26.
